# Multi-omic spatial profiling reveals the unique SARS-CoV-2 lung microenvironment and collagen VI as a predictive biomarker in severe COVID-19

**DOI:** 10.1183/13993003.01699-2023

**Published:** 2025-09-11

**Authors:** Éanna Fennell, Graham S. Taylor, Ciara I. Leahy, Aisling M. Ross, Gary Reynolds, Tracey Perry, Esther Youd, Jacob Skidmore, Radwan Ramzi Radwan Darwish, Kelly J. Hunter, Benjamin E. Willcox, Philip Jermann, Chowdhury Arif Jahangir, Arman Rahman, William M. Gallagher, Nadezhda Nikulina, Bassem Ben Cheikh, Oliver Braubach, Aaron T. Mayer, Lawrence S. Young, Dimitris Grammatopoulos, Sian Faustini, Alex Richter, Alexander C. Dowell, Tonny Venith, Onn S. Thein, Dhruv Parekh, Kylie B.R. Belchamber, David R. Thickett, Aaron Scott, Richard Attanoos, Lucia Mundo, Stefano Lazzi, Lorenzo Leoncini, Gareth Leopold, Neil Steven, Jannie Marie Bülow Sand, Morten A. Karsdal, Diana Julie Leeming, Stefan Dojcinov, Aedin Culhane, Paul G. Murray, Matthew R. Pugh

**Affiliations:** 1 School of Medicine, University of Limerick, Limerick, Ireland; 2 Bernal Institute, University of Limerick, Limerick, Ireland; 3 Limerick Digital Cancer Research Centre, Health Research Institute, University of Limerick, Limerick, Ireland; 4 Department of Immunology and Immunotherapy, School of Infection, Inflammation and Immunology, College of Medicine and Health, University of Birmingham, Birmingham, UK; 5 Forensic Medicine and Science, University of Glasgow, Glasgow, UK; 6 School of Medicine, Royal College of Surgeons in Ireland – Medical University of Bahrain, Adliya, Bahrain; 7 Birmingham Tissue Analytics, University of Birmingham, Birmingham, UK; 8 Department of Medical Genetics and Pathology, University Hospital Basel, Basel, Switzerland; 9 School of Biomolecular and Biomedical Science, University College Dublin, Dublin, Ireland; 10 Institute of Lung Health, Justus Liebig University Giessen, Giessen, Germany; 11 Akoya Biosciences, Marlborough, MA, USA; 12 Bruker Spatial Biology, St Louis, MO, USA; 13 Enable Medicine, Menlo Park, CA, USA; 14 Warwick Medical School, University of Warwick, Coventry, UK; 15 Clinical Immunology Service, University of Birmingham, Birmingham, UK; 16 Department of Critical Care and Anaesthesia, Queen Elizabeth Hospital Birmingham, Birmingham, UK; 17 Birmingham Acute Care Research Group, Institute of Inflammation and Ageing, University of Birmingham, Birmingham, UK; 18 Department of Pathology, University Hospital of Wales, Cardiff, UK; 19 Section of Pathology, Department of Medical Biotechnologies, University of Siena, Siena, Italy; 20 Department of Cellular Pathology, Swansea Bay University Health Board, Swansea, UK; 21 Nordic Bioscience, Herlev, Denmark; 22 Department of Data Science, Dana-Farber Cancer Institute, Boston, MA, USA; 23 Department of Biostatistics, Harvard T.H. Chan School of Public Health, Boston, MA, USA; 24 É. Fennell and G.S. Taylor contributed equally to this work; 25 P.G. Murray and M.R. Pugh contributed equally to this work

## Abstract

**Background:**

While coronavirus disease 2019 (COVID-19) is primarily a respiratory infection, few studies have characterised the immune response to COVID-19 in lung tissue. We sought to understand the pathogenic role of microenvironmental interactions and the extracellular matrix in post-mortem COVID-19 lung using an integrative multi-omic approach.

**Methods:**

Post-mortem formalin-fixed paraffin-embedded lung tissue from fatal COVID-19 and nonrespiratory death control lung underwent multi-omic evaluation by Quantseq Bulk RNA sequencing, Nanostring GeoMx spatial transcriptomics, RNAscope, multiplex immunofluorescence and immunohistochemistry, to evaluate virus distribution, immune composition and the extracellular matrix. Markers of extracellular synthesis and breakdown were measured in the serum of 215 patients with COVID-19 and 54 healthy volunteer controls using ELISA.

**Results:**

We found that severe acute respiratory syndrome coronavirus 2 (SARS-CoV-2) infection was restricted to the pneumocytes and macrophages of early-stage disease. Spatial analyses revealed an immunosuppressive virus microenvironment, enriched for PDL1^+^IDO1^+^ macrophages and depleted of T-cells. Oligoclonal T-cells in COVID-19 lung showed no enrichment of SARS-CoV-2 specific T-cell receptors. Collagen VI was upregulated and contributed to alveolar wall thickening and impaired gas exchange in COVID-19 lung. Serum from COVID-19 patients showed increased levels of PRO-C6, a marker of collagen VI synthesis, predicted mortality in hospitalised patients.

**Conclusions:**

Our data refine the current model of respiratory COVID-19 with regard to virus distribution, immune niches and the role of the noncellular microenvironment in pathogenesis and risk stratification in COVID-19. We show that collagen deposition is an early event in the course of the disease.

## Shareable abstract

**The SARS-CoV-2 lung microenvironment is enriched for immunosuppressive macrophages in severe disease. Collagen VI deposition contributes to alveolar thickening, and detection of serum PRO-C6 is predictive for mortality in hospitalised COVID-19 patients.** https://bit.ly/3E3Wh1s

## Graphical abstract

**Figure:**
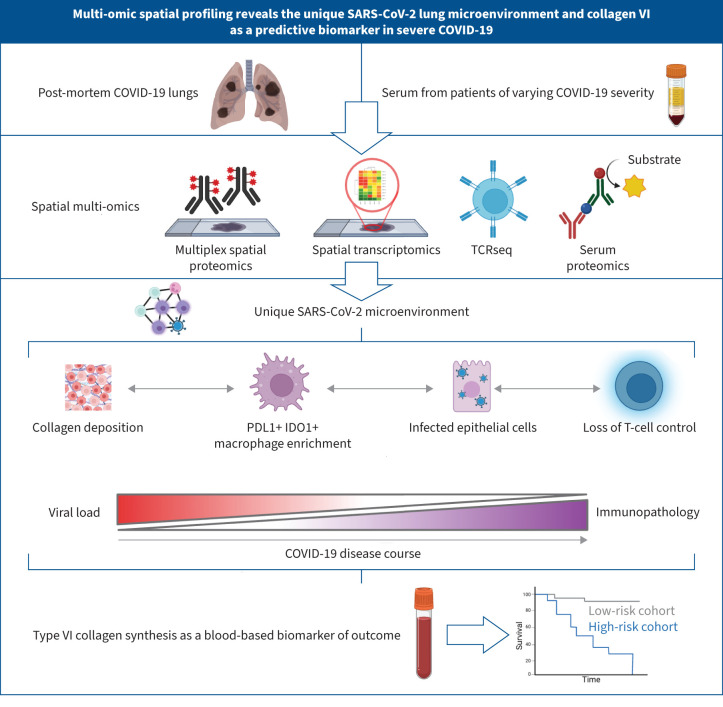
Overview of the study. SARS-CoV-2: severe acute respiratory syndrome coronavirus 2; COVID-19: coronavirus disease 2019; TCR: T-cell receptor.

## Introduction

Although vaccination efforts have reduced mortality from coronavirus disease 2019 (COVID-19) [1], detailed insights into virus–host interactions in infected tissues are required to develop better therapies for people who are ineligible for, or respond poorly to, vaccination [2–4]. Knowledge of the mechanisms underpinning the development of severe COVID-19 could also identify biomarkers to better stratify patients.

While changes in the innate and adaptive immune responses to severe acute respiratory syndrome coronavirus 2 (SARS-CoV-2) infection have been characterised extensively in the blood [5], understanding the true nature of respiratory COVID-19 requires characterisation of immunopathology in lung tissues. Bronchoalveolar lavage (BAL) studies have helped characterise the alveolar compartment [6, 7], but cannot fully describe the pathology of other lung components, such as the interstitium and blood vessels, which are poorly represented in BAL samples [8]. Furthermore, studies of dissociated lung tissues [9, 10] lack information on spatial relationships [11] and while the histology of SARS-CoV-2-infected lung is well described [12–14], the complexity of the immune landscape has not been properly characterised [15]. Defining the immune microenvironment of virus-infected cells has been particularly challenging, because of conflicting reports on which cells are infected [6, 9, 16, 17]. Moreover, collagen deposition, recognised for its role in COVID-19 lung fibrosis, is upregulated in the acute phase, but its clinical significance is unknown [15, 18–20].

To address these shortcomings, we have conducted a deep spatial immunopathological characterisation of COVID-19-infected lungs, describing the immune landscape, the nature and microenvironment of infected cells, and the dynamics of extracellular matrix deposition. We report the identification of new serological biomarkers of mortality which could be used to identify high-risk patients and guide better patient management.

## Materials and methods

Multi-omic profiling was initially conducted on formalin-fixed paraffin-embedded (FFPE) lung tissues from eight fatal COVID-19 cases from South Wales, UK (April–August 2020) and controls, including Middle East respiratory syndrome (MERS) (n=1), rhinovirus (n=1), bacterial pneumonia (n=3) and noninfectious samples (n=8). Validation cohorts included tissues from nine COVID-19 cases and three nonrespiratory deaths from London, UK, and 10 COVID-19 cases from Siena, Italy. Serum samples were obtained from healthy controls and COVID-19 patients described in detail later.

Tissues were stained with haematoxylin and eosin (H&E) and picrosirius red (PSR). Chromogenic immunohistochemistry for collagen VI was performed on the Leica BondMax. A 38-plex multiplex immunofluorescence (mIF) panel against immune lineage, functional and viral markers (supplementary data file S1b) was performed on a tissue microarray using the PhenoCycler FUSION platform. A 10-plex focused panel was applied to a wider collection of lung tissues (supplementary data file S1b). A separate 21-plex antibody validation panel was performed on the Lunaphore COMET to identify collagens, structural markers, SARS-CoV-2 and immune cells.

Spatial transcriptomics was performed on three SARS-CoV-2 lungs, one MERS lung, one rhinovirus lung, one bacterial pneumonia lung and two normal lungs (supplementary data file S1a) using the Nanostring GeoMx Digital Spatial Profiler (DSP) and the Cancer Transcriptome Atlas panel with SARS-CoV-2-specific spike-in probes.

Bulk RNA sequencing (RNAseq) was performed using the Lexogen QuantSeq 3′ mRNA-Seq Kit FWD for Illumina sequencing. SARS-CoV-2 and interleukin (IL)6 mRNA was detected by *in situ* RNAscope and Leica Multiplex Fluorescent Assay using probes against sense strands of the *spike* gene (V-nCoV2019-S, 845701, ACD), antisense strand of the *orf1ab* gene (V-nCoV2019-ORF1ab-sense, ACD) and human *IL6* (ACD).

T-cell receptor (TCR) sequencing was performed using the Oncomine TCR Beta-LR Assay (Thermo Fisher), with preparation on the Ion Chef System and sequencing on the Ion GeneStudio S5 system.

10 markers of extracellular matrix formation and degradation and a marker of neutrophil activation (supplementary table S10) were measured by competitive ELISA (Nordic Bioscience; Herlev Denmark) in the serum of healthy controls and COVID-19 patients of varying disease severity (community, convalescent, acute hospital, acute intensive therapy unit (ITU) and convalescent ITU).

## Results

### Multi-omic analysis of post-mortem lung tissues

**FIGURE 1:**
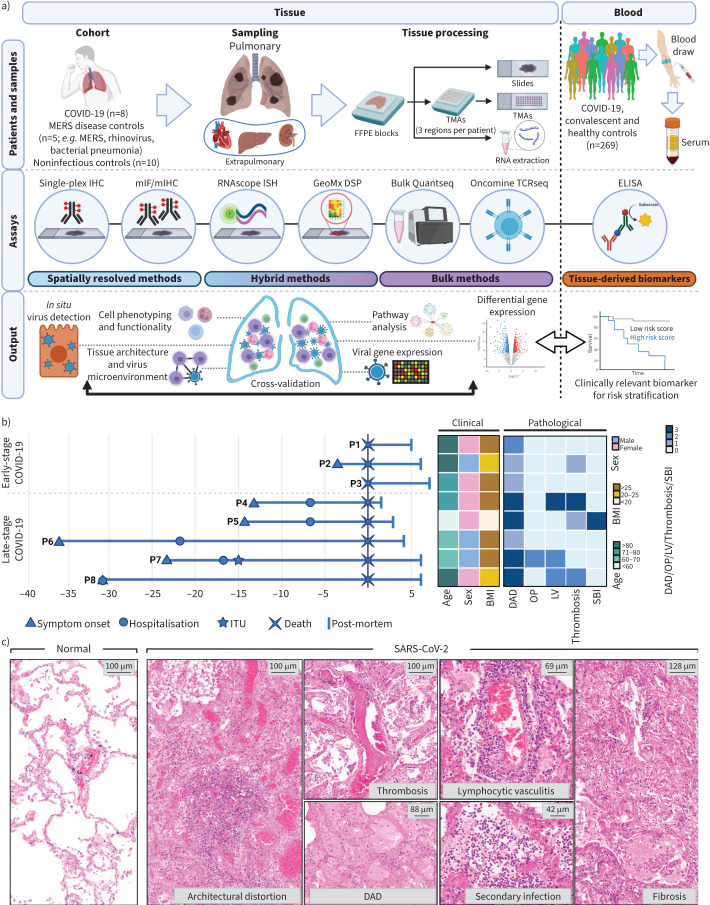
Study design and temporal-clinicopathological features of tissue cohort. a) Schematic diagram of study workflow; b) disease timeline of coronavirus disease 2019 (COVID-19) cohort with associated clinical and pathological information; c) representative haematoxylin and eosin images of normal and COVID-19 lung tissue. MERS: Middle East respiratory syndrome; FFPE: formalin-fixed paraffin-embedded; TMA: tissue microarray; IHC: immunohistochemistry; mIF: multiplex immunofluorescence; ISH: *in situ* hybridisation; DSP: Digital Spatial Profiler; ITU: intensive therapy unit; BMI: body mass index; DAD: diffuse alveolar damage; OP: organising pneumonia; LV: lymphocytic vasculitis; SBI: secondary bacterial infection.

Following histopathological assessment, FFPE lung tissues from eight patients who died from COVID-19 during the first pandemic wave, as well as pre-pandemic controls, comprising people who had died from nonrespiratory causes (n=5), bacterial pneumonia (n=1), rhinovirus pneumonia (n=1) and MERS (n=1) were subjected to 1) chromogenic single-plex immunohistochemistry, 38-marker mIF (PhenoCycler FUSION) targeting immune lineage markers and SARS-CoV-2 (supplementary data file S1b), and 21-marker multiplex immunohistochemistry on the COMET platform for immune lineage and extracellular matrix components; 2) multiplex RNAscope for SARS-CoV-2 RNA and pro-inflammatory cytokines; 3) Nanostring GeoMx DSP for alveolar and blood vessel tissue compartments (supplementary figure S1); 4) whole-transcriptome bulk mRNA expression by Quantseq 3′ RNAseq; and 5) Oncomine TCR-β chain sequencing to define T-cell receptor specificities (figure 1a, supplementary table S1, supplementary data file S1a). Validation was performed on two separate post-mortem cohorts comprising lung tissues from nine COVID-19 cases and three controls (Imperial College London Tissue Bank, UK), and 10 COVID-19 cases (University of Siena, Italy).

### Fatal COVID-19 shows a variable clinical course and ubiquitous diffuse alveolar damage

Of the initial eight patients (all white British; three males, five females; mean age 74 years, range 57–89 years, mean body mass index 26 kg·m^−2^ (figure 1b and supplementary table S1), three died in the community early in the course of COVID-19 (0–3 days from symptoms to death; early-stage), while the remaining five patients died in hospital following a more protracted illness (13–36 days from symptoms to death; late-stage). Positive SARS-CoV-2 virus swabs were obtained at post-mortem for the three community deaths, and in life for the five hospital deaths. Of the latter, one patient underwent noninvasive ventilation, and one was admitted to intensive care with invasive ventilation. Pathological assessment identified diffuse alveolar damage in all patients. One patient showed florid organising pneumonia. A subset of cases showed variable degrees of microthrombi (four out of eight), lymphocytic vasculitis (three out of eight) and secondary bacterial/fungal infection (five out of eight) (figure 1b, c and supplementary table S1). The two validation COVID-19 cohorts were from patients with late-stage disease (>7 days symptom onset to death) (supplementary data file S1h, i) and all showed diffuse alveolar damage (supplementary data file S1h, i).

### SARS-CoV-2 infection is confined to lung epithelium and a minority of macrophages in respiratory COVID-19

**FIGURE 2:**
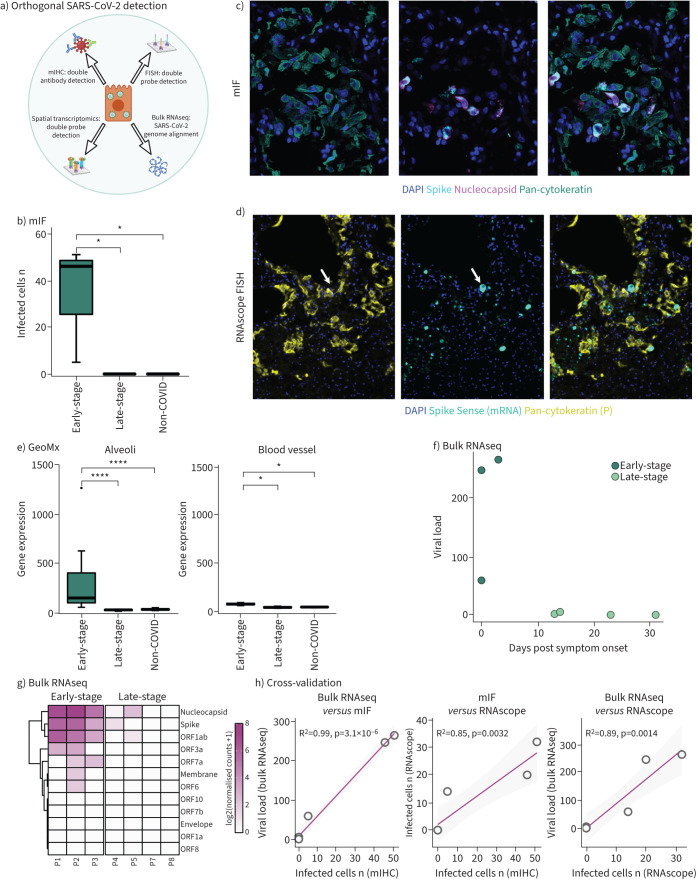
Severe acute respiratory syndrome coronavirus 2 (SARS-CoV-2) detection, distribution, features and validation. a) Schematic of multi-omic SARS-CoV-2 detection; b) quantification of SARS-CoV-2-infected cells; c) representative multiplex immunohistochemistry (mIHC) images of SARS-CoV-2 dual antibody detection; d) representative RNAscope fluorescence *in situ* hybridisation (FISH) images of SARS-CoV-2 infection in macrophages; e) localisation of SARS-CoV-2 infection predominantly to the alveoli by Nanostring GeoMx Digital Spatial Profiler (DSP); f) bulk RNA sequencing (RNAseq) viral load as a function of days post symptom onset of coronavirus disease 2019 (COVID-19); g) breakdown of viral gene expression across early- and late-stage COVID-19 by bulk RNAseq; h) cross-platform validation of SARS-CoV-2 detection (mIHC, RNAscope FISH and bulk RNAseq). mIF: multiplex immunofluorescence; DAPI: 4',6-diamidino-2-phenylindole. *: p<0.05; ****: p<0.0001.

We used a robust multi-omic approach to identify SARS-CoV-2 (figure 2a, supplementary figure S1). The initial mIF-based detection of SARS-CoV-2 required the colocalisation of both spike and nucleocapsid proteins, which are expressed contemporaneously during infection (supplementary figure S1) [21, 22]. Automatic histogram thresholding revealed that infected cells were present only in early-stage patients (*i.e.* died within the first 3 days following symptom onset) (figure 2b). Almost all virus-positive cells had type-2 pneumocyte morphology, were localised to alveoli, and expressed pan-cytokeratin (figure 2c). A small fraction of CD68-positive macrophages were positive (supplementary figure S2). We did not detect infection of any other cell type. We used RNAscope to confirm these findings and observed SARS-CoV-2 genomic RNA only in epithelial cells from early-stage patients (figure 2d). Spatial transcriptomics by Nanostring GeoMx confirmed the high levels of *spike* and *orf1ab* mRNA in the alveolar compartment, with only small numbers of viral reads in the vascular compartment (figure 2e). Bulk RNAseq showed that virus gene expression in early disease was dominated by expression of *nucleocapsid*, *spike* and *orf1ab* genes, although very low numbers of virus reads remained in some late-stage patients (figure 2f, g). Bulk RNAseq performed on the validation cohort of late-stage COVID-19 lungs showed very low SARS-CoV-2 virus (zero to five reads) in eight out of nine cases and high virus (106 reads) in one case, consistent with early virus clearance; this latter patient died 8 days after symptom onset (supplementary data file S1j). Collectively, our robust virus detection methodology showed excellent concordance and demonstrated that SARS-CoV-2 virus is localised predominantly to lung epithelium and a minority of macrophages (figure 2h).

### COVID-19 lungs are infiltrated with diverse immune cell types, but are not enriched for SARS-CoV-2 specific T-cells

To assess cell frequency, deconvolution was performed on the Nanostring GeoMx DSP gene expression data (supplementary figure S3). As expected, cases of bacterial pneumonia showed prominent neutrophil infiltration. COVID-19 lungs showed mixed populations with a predominance of macrophages/monocytes, neutrophils and fibroblasts. Smaller populations of CD4^+^ T-cells, CD8^+^ T-cells, natural killer (NK) cells, plasma cells and B-cells were also present. Notably, CD4^+^ T-cells were proportionally greater in normal and rhinovirus controls.

**FIGURE 3:**
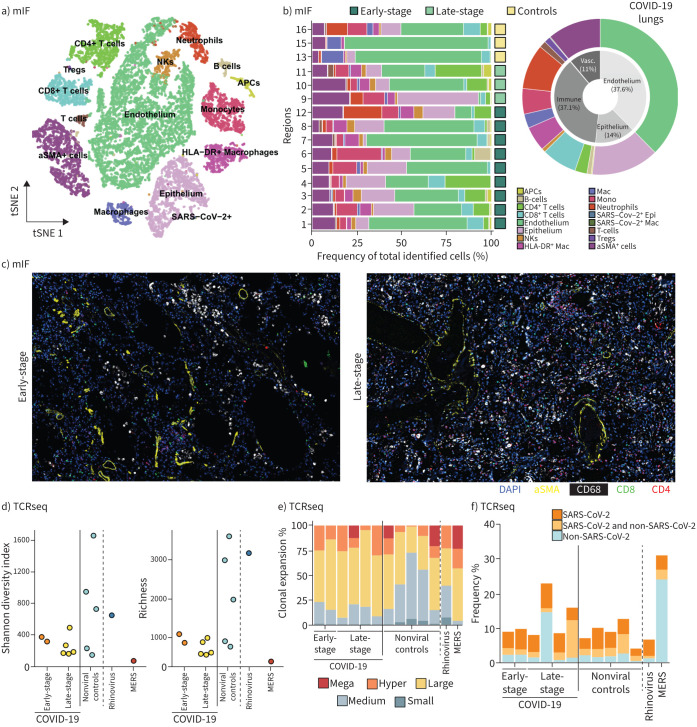
Immune landscape and T-cell repertoire of coronavirus disease 2019 (COVID-19) lung infection. a) Downsampled t-distributed stochastic neighbour embedding (tSNE) plot of identified cell types by multiplex immunohistochemistry (mIHC); b) cellular abundances of identified phenotypes per region analysed and pie chart of cell types present in COVID-19 lungs; c) representative images of the immune shift from early- to late-stage disease; d) Shannon diversity and richness of the COVID-19 tissue T-cell receptor (TCR) repertoire; e) clonal expansions of the tissue TCR repertoire; f) specificity of the tissue TCR repertoire. NK: natural killer; SMA: smooth muscle actin; Tregs: regulatory T-cells; APC: antigen-presenting cell; SARS-CoV-2: severe acute respiratory syndrome coronavirus 2; HLA: human leukocyte antigen; Mac: macrophage; Mono: monocyte; Epi; epithelial; MERS: Middle East respiratory syndrome; DAPI: 4',6-diamidino-2-phenylindole.

To characterise the distribution of immune cells, a subset of four COVID-19 cases and three controls were subject to 38-plex mIF (supplementary data file S1b). Images were de-arrayed and segmented with CellSeg [23], and the resulting ∼400 000 cells analysed by CELESTA to identify major cell types (supplementary figure S3), including epithelial cells (pan-cytokeratin^+^), endothelial cells (CD34^+^), immune cells (CD45^+^) and smooth muscle actin-expressing mesenchymal cells (aSMA^+^) [24]. Immune cells were clustered with PhenoGraph to identify specific subpopulations (supplementary figure S3) [25]. The total number of cells were downsampled to ∼40 000 and visualised on a t-distributed stochastic neighbour embedding plot (figure 3a). In COVID-19 lungs, the predominant immune populations were monocytes/macrophages, neutrophils, CD4^+^ T-cells and CD8^+^ T-cells, with smaller numbers of NK cells and B-cells (figure 3b). Macrophages, neutrophil and T-cells showed a trend towards increasing numbers in late-stage disease cases compared to early-stage, despite clearance of the virus from late-stage cases (figure 3c, supplementary figure S3). Analysis of bulk sequencing data comparing differential expression in early-stage and late-stage COVID-19 lungs showed an upregulation of *MX1*, an interferon-mediated viral response gene in early-stage disease (supplementary figure S3). This supports a viral mediated immune early response followed by viral clearance and ongoing immune infiltration.

To explore the specificity of the infiltrating T-cells, we performed short-read TCR sequencing from seven of the eight COVID-19 lung FFPE samples and seven control patients including the rhinovirus and MERS pneumonia cases (supplementary data file S1c). All seven COVID-19 cases, regardless of disease stage, and the single MERS case had similarly low values of Shannon index of diversity and richness (figure 3d), indicating oligoclonal TCR sequences. Accordingly, every COVID-19 lung sample, and the MERS lung sample, contained TCR sequences that comprised 0.1–1% of the total repertoire, 1–10% of the total repertoire and, for two late-stage COVID-19 cases and the MERS case, >10% of the repertoire (figure 3e). We explored the antigen specificity of the TCR-β sequences in all lung samples by comparing them to sequences reported to be specific for SARS-CoV-2 [26–30]. Although every COVID-19 lung sample contained TCR-β sequences matching those reported to be SARS-CoV-2 specific, we detected similar proportions of matching TCR-β sequences in the control non-COVID-19 cases (figure 3f). TCR sequences reported to be specific for other pathogens, including influenza, Epstein–Barr virus (EBV) and cytomegalovirus (CMV) were also present, and in some cases comprised a substantial portion of the repertoire (supplementary figure S4).

### The microenvironment of SARS-CoV-2-infected cells is enriched for immunosuppressive macrophages and depleted of T-cells

**FIGURE 4:**
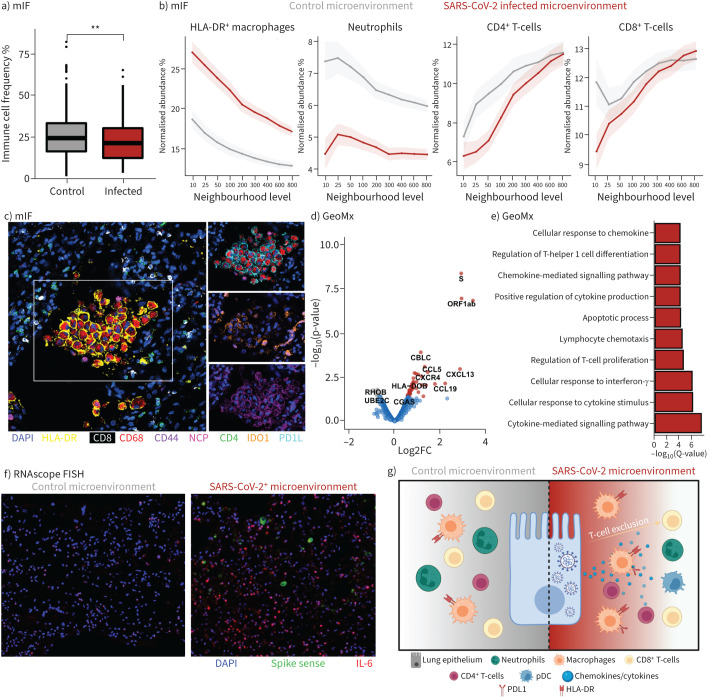
Immune and inflammatory virus microenvironment of severe acute respiratory syndrome coronavirus 2 (SARS-CoV-2). a) Immune cell abundance in the microenvironment of infected and control epithelium; b) immune cell abundance as a function of distance from SARS-CoV-2 infected epithelium and control epithelium; c) representative images and segmentation mask of SARS-CoV-2 microenvironment; d) differential gene expression of SARS-CoV-2-high *versus* -low alveoli by Nanostring GeoMx Digital Spatial Profiler (DSP); e) GO pathway analysis of SARS-CoV-2-positive alveoli; f) interleukin (IL)-6 expression by RNAscope in SARS-CoV-2-positive microenvironments; g) model of immune control and evasion in the SARS-CoV-2 microenvironment. mIF: multiplex immunofluorescence; HLA: human leukocyte antigen; DAPI: 4',6-diamidino-2-phenylindole; FISH: fluorescence *in situ* hybridisation; IL: interleukin; pDC: plasmacytoid dendritic cell.

We re-analysed the mIF data, measuring immune cell abundance in the 100 cells nearest to virus-infected epithelial cells compared to noninfected epithelial cells of the same cases. We found that overall immune cell (CD45^+^ cells identified by CELESTA) numbers were significantly reduced in the proximity of SARS-CoV-2-infected epithelial cells (figure 4a). However, against this background of an overall immune cell deficit, we found that the numbers of human leukocyte antigen-DR^+^ macrophages were significantly increased irrespective of their distance from SARS-CoV-2-infected epithelial cells (figure 4b, c). Moreover, most of these macrophages co-expressed inhibitory ligands, PDL1 and IDO1 (figure 4c and supplementary figure S5). In contrast, neutrophils were significantly depleted from SARS-CoV-2-positive microenvironments (figure 4b). CD4^+^ and CD8^+^ T-cells were also depleted from regions immediately adjacent to infected cells (figure 4b). To explore possible mechanisms responsible for these differences, we profiled gene expression in the alveolar microenvironment by Nanostring GeoMx DSP (figure 4d and supplementary data file S1g). Alveolar regions from the same patient were assigned as either virus-high or virus-low based on the levels of *spike* and *orf1ab* mRNA (supplementary figure S5). Virus-high alveoli displayed higher expression of numerous chemokines and chemokine receptors (*e.g. CCL5*, *CXCL13*, *CXCR4*, *CCL19*, *CCL2*, *CXCL11*), interferon-α (*IFNA1*) and interferon regulatory factors (*e.g. IRF8*, *IRF1*). Pathway analysis showed enrichment of chemokine/cytokine and lymphocyte chemoattractant pathways among upregulated genes from SARS-CoV-2-infected microenvironments (figure 4e). Furthermore, RNAscope fluorescence *in situ* hybridisation showed enrichment of *IL-6*-expressing cells in the microenvironment of SARS-CoV-2-infected cells compared to noninfected regions of the same tissues (figure 4f). Thus, T-cells are excluded from the immediate microenvironment of SARS-CoV-2-infected epithelial cells despite high levels of lymphocyte chemoattractants (figure 4g).

### Expression of type VI collagen is an early event in the pathogenesis of fatal respiratory COVID-19 and MERS

**FIGURE 5:**
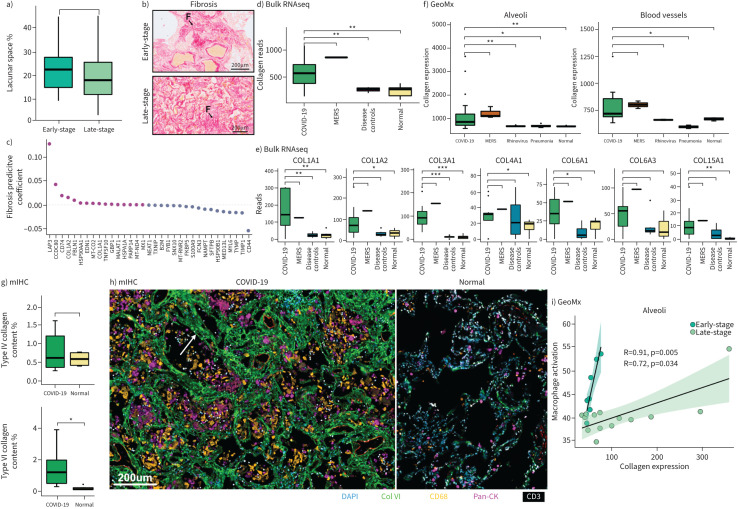
Collagen landscape of fatal coronavirus disease 2019 (COVID-19). a) Lacunar space quantification; b) representative picosirius red images from early- and late-stage COVID-19; c) fibrotic predictive coefficient of genes; d) tissue-wide transcription of collagens in COVID-19 lungs and controls; e) tissue-wide transcription of type 1, 3, 4, 6 and 15 collagens in COVID-19 lungs on controls; f) spatially resolved collagen gene expression to alveoli and blood vessels; g) quantification of type 4 and 6 collagen deposition from multiplex immunohistochemistry (mIHC); h) representative images of type 6 collagen deposition in COVID-19 lungs by COMET mIHC. Scale bar=200 μm; i) correlation of collagen gene expression and macrophage activation. F: fibrosis; MERS: Middle East respiratory syndrome; DAPI: 4',6-diamidino-2-phenylindole. *: p<0.05; **: p<0.001; ***: p<0.001.

Several collagen genes contributing to lung fibrosis have been reported to be upregulated in COVID-19 lungs [10, 15, 31]. To assess fibrosis-associated changes we first quantified alveolar wall thickness on H&E stains. This revealed a reduction in mean alveolar space in patients who had a longer disease duration compared to those who had died early after symptom onset (figure 5a, supplementary figure S6). In addition, we stained tissues with picrosirius red (figure 5b) which revealed extensive fibrosis in both early- and late-stage COVID-19 patients (supplementary figure S6). Fitting a least absolute shrinkage and selection operator regression model between these morphological features and bulk RNAseq identified collagen subunits (*COL1A1* and *COL1A2*), heat shock proteins (*HSP90AA1* and *HSPA1A*) and a cytosolic leucine aminopeptidase involved in antigenic protein processing (*LAP3*) as the genes most predictive of fibrosis (figure 5c). *COL1A1* and *COL1A2* were among the genes most predictive of lacunar space reduction (supplementary figure S6).

Similarly, bulk RNA sequencing analysis revealed the upregulation of collagen genes, including *COL1A1*, *COL1A2*, *COL3A1*, *COL4A1*, *COL6A1*, *COL6A3* and *COL15A* (figure 5d, e). The same collagens were also upregulated in the single case of MERS pneumonia, but not in the rhinovirus or bacterial pneumonia lungs. Nanostring GeoMx spatial analysis also showed the upregulation of collagens in the alveolar compartment of the late-stage COVID-19 and MERS (figure 5f, supplementary figure S6). These findings were confirmed by bulk Quantseq RNAseq in a separate cohort of post-mortem lungs (eight COVID-19; three deaths from nonrespiratory causes) which revealed the upregulation of *COL1A1*, *COL1A2*, *COL3A1*, *COLA41*, *COL6A1* and *COL6A3* in COVID-19 lung compared to controls (supplementary figure S6e).

To assess the spatial distribution of collagens at a proteomic level, we established a 21-plex antibody panel on the Lunaphore COMET platform targeting collagen type I, III, IV and VI and cell lineage markers (supplementary data file S1b). Collagen type VI was markedly increased in the alveolar interstitium compared to normal controls (figure 5g, h, supplementary figure S6). In the alveolar walls, collagen type VI showed a fibrillar distribution and colocalised with alveolar wall thickening. Increased collagen VI in SARS-CoV-2 lung compared to controls was confirmed upon quantitative assessment (p<0.05) (figure 5g). Collagens I, III and IV did not show a marked increase in COVID-19 lungs compared to normal controls.

To confirm the increased interstitial deposition of collagen VI in COVID-19 lungs, two validation cohorts comprising post-mortem lung tissues from the UK and Italy were stained for collagen VI by chromogenic immunohistochemistry. Both cohorts showed an increase in the collagen to alveolar wall ratio contributing to alveolar wall thickening and respiratory dysfunction (supplementary figure S6h, i). The increase was statistically significant in the Italian cohort (p=0.007). Of note, the mean time from symptom onset to death was longer in the Italian cohort (18 days, range 9–47 days) compared to UK patients (13 days, range 8–24 days) (supplementary data file S1h, i).

Because macrophages are important in the fibroblast-mediated deposition of collagen in the lung, we compared macrophage activation and collagen expression [32] by plotting total alveolar expression of a macrophage activation gene set against the summated expression of collagens, *COL1A*, *COL3A*, *COL4A* and *COL6A* (figure 5i). Splitting the results into early- and late-stage patients, we found a strong correlation between macrophage activation and collagen expression in both disease groups (early-stage R=0.91, p=0.005; late-stage R=0.72, p=0.0034).

### Elevated serum PRO-C6, a biomarker of type VI collagen synthesis, defines high-risk COVID-19

**FIGURE 6:**
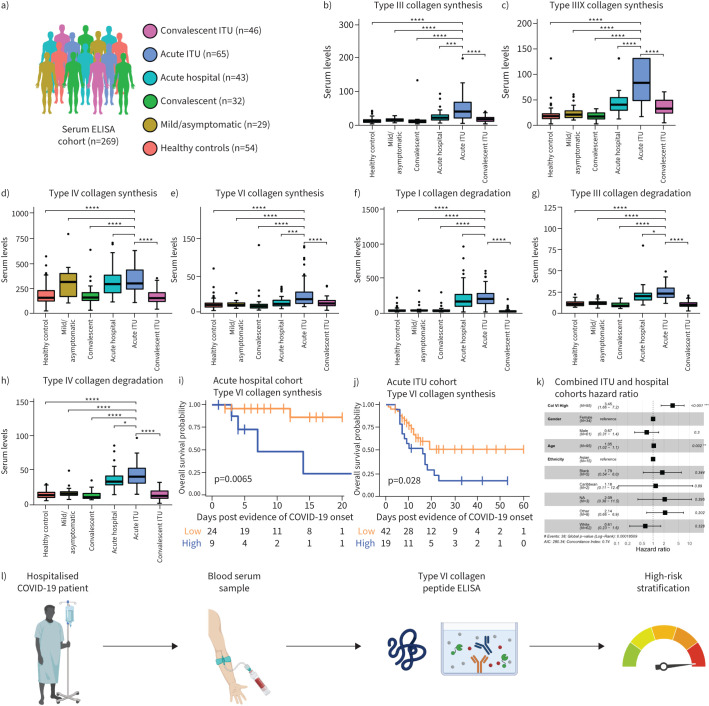
Blood detection of tissue-derived extracellular matrix landscape of coronavirus disease 2019 (COVID-19). a) Serum proteomics cohort description; b–h) collagen formation and degradation peptide serum levels for each group; i) survival cures for acute hospital patients based on serum collagen VI; j) survival cures for intensive therapy unit (ITU) patients based on serum collagen VI; k) demographic variable hazard ratios for outcome; l) blood-based biomarker of tissue derived collagen formation peptides for patient risk stratification. *: p<0.05; **: p<0.001; ***: p<0.001; ****: p<0.0001.

We measured collagen synthesis and degradation using ELISA in the serum of 215 COVID-19 patients of varying severity (mild/asymptomatic, acute hospital, acute ITU, convalescent and convalescent ITU; figure 6a) and 54 healthy controls. Markers of synthesis of collagen types III (PRO-C3) and VI (PRO-C6), crosslinked type III collagen (PC3X), and degradation of collagen types I (C1M), III (C3M) and VI (C6M) were all significantly elevated in acutely hospitalised cases, including those in ITU, but were decreased in convalescing patients (figure 6b, c, e). A marker of type IV collagen synthesis (PRO-C4) was significantly increased in acute ITU cases compared to healthy and convalescent groups, but not compared to mild/asymptomatic or acute hospital COVID-19 groups (figure 6d). Furthermore, blood clot formation (PRO-FIB) and resolution (X-FIB), measured through circulating fibrinogen peptides, as well as two markers related to neutrophil activity (CPa9-HNE, ELP-3), were also increased in acute cases (supplementary figure S7).

Acute hospital and acute ITU patients were stratified into high or low groups according to the mean serum level of each marker. Patients with high PRO-C6 had significantly worse overall survival (acute hospital p=0.0065; acute ITU=0.028) (figure 6i, j). No other serum extracellular matrix peptide showed a significant association with survival. Combining both acute hospital and acute ITU cohorts, the high PRO-C6 group had poorer survival compared to low PRO-C6 patients, after adjusting for age, sex and ethnicity (hazard ratio 3.45, p<0.001) (figure 6k, supplementary figure S7). Our results identify PRO-C6 as a potential noninvasive test to identify high-risk hospitalised COVID-19 patients (figure 6l).

## Discussion

Using complementary multi-omic spatial and genomic assays, we profiled the immunopathology of respiratory COVID-19. Our findings provide new information on the nature of infected cell types, the host response and the impact of duration of infection, including changes in extracellular matrix composition. In addition, we have described the immediate immune microenvironment of virus-infected cells identifying several immune-evasive mechanisms. Finally, our data reveal a serum-based biomarker that identifies high-risk patients.

Autopsy specimen analysis requires robust validation due to tissue autolysis and nucleic acid degradation following FFPE sample processing. Our data were highly concordant across different multi-omic assays and showed that SARS-CoV-2 was detectable mainly only in those who died early in the disease course. Although bulk sequencing detected trace amounts of viral RNA in patients with more advanced disease, viral proteins were undetectable. This time course is consistent with previous studies analysing fatal cases of COVID-19 that showed the virus is cleared from the lung 2 weeks after symptom onset [33–36]. The range of cell types in the lung reported to be infected by SARS-CoV-2 varies between studies. Some detected the virus only in epithelial cells and macrophages [6, 35, 37] while others report that the virus can infect a much wider range of immune cells, including neutrophils, mast cells, B-cells and T-cells [9, 15]. Based on our stringent requirement for colocalisation of viral spike and nucleocapsid proteins we detected SARS-CoV-2 only in epithelial cells and a small number of macrophages. Macrophages can be infected with SARS-CoV-2 *in vitro* and although this infection is abortive, the ensuing inflammasome activation, cytokine production and pyroptosis appear to be strong contributors to the profound pro-inflammatory state [37, 38].

Our combined spatial transcriptomic and proteomic analysis allowed us to investigate the immune neighbourhood of the SARS-CoV-2-infected cells and how this differed compared to noninfected epithelial cells in the same tissues. Like other studies, we observed prominent intra-alveolar macrophages and an interstitial lymphocytic infiltrate predominantly comprising T-cells [7, 39]. However, the immediate microenvironment of infected epithelial cells was depleted of both CD4^+^ and CD8^+^ T-cells, potentially limiting the effectiveness of adaptive antiviral immunity. Unexpectedly, while our TCR sequencing data detected oligoclonal expanded T-cells, lung tissues from COVID-19 patients were not enriched for TCR sequences reported to be SARS-CoV-2-specific [27–29]. We detected substantial numbers of TCR sequences reported to be specific for nonrespiratory viruses such as CMV and EBV in COVID-19 and control lung tissues. Therefore, a large proportion of T-cells in the lungs of patients who died from COVID-19, rhinovirus or MERS appear to not be specific for the pathogenic infectious agent and are instead presumably recruited nonspecifically in response to chemokine signals. Because we only studied fatal cases of infection, we cannot exclude the possibility that larger numbers of SARS-CoV-2-specific T-cells are present in those who recover. In contrast, macrophages were enriched in the neighbourhoods of infected cells and expressed immunosuppressive molecules including PDL1 and IDO-1, most likely induced by the strong interferon signature observed in infected alveoli [40–42].

Increased collagen synthesis plays a key role in chronic fibrotic lung conditions, such as idiopathic pulmonary fibrosis (IPF) [43, 44]. Our data suggest that collagen VI contributes to acute respiratory dysfunction through thickening of the alveolar wall, thus impeding gas exchange. The increased expression of collagens and markers of collagen synthesis in patients with more advanced COVID-19 suggests that collagen deposition is an ongoing process rather than a result of pre-existing lung fibrosis. Collagen gene expression was also upregulated in the single case of MERS pneumonitis but not in cases of rhinovirus or bacterial pneumonia. Other studies have shown that collagens are not upregulated in the lungs in acute influenza infection [31]. Thus, increased collagen upregulation may be a feature of the acute phase of betacoronoavirus infections. Rhesus macaques infected with SARS-CoV-2 exhibit mild disease, in contrast to humans. Notably, collagen gene expression is downregulated in their lungs, suggesting a potential link between the absence of collagen upregulation and reduced disease severity in this model [45].

Whether collagen expression drives disease severity remains unclear. Collagen upregulation in COVID-19 may result from SARS-CoV-2 binding to angiotensin converting enzyme 2, disrupting the renin–angiotensin system and relieving the suppression of fibrosis [46]. This mechanism could explain early collagen deposition during acute infection. A similar pro-fibrotic role has been proposed for DPP4, the receptor of MERS coronavirus [47]. Macrophages may also play a role in driving collagen upregulation and fibrosis. In severe COVID-19 patients with acute respiratory disease syndrome, macrophages upregulate pro-fibrotic pathways and have a phenotype like those found in IPF [31]. Macrophages in COVID-19 and IPF upregulate genes that induce fibrosis and activate pro-fibrotic fibroblasts (*e.g.* COL, FGF, SPP1, TGFB1, TGFBI, LGMN and CCL18). Our data align with these findings, showing that macrophage activation is strongly associated with collagen upregulation. Similarly, MERS infects macrophages in humans and animal models, rapidly increasing lung collagen expression [48, 49].

We demonstrate that collagen upregulation measured using simple serum-based assays is associated with disease severity and outcome in the acute phase of the disease. Such a biomarker could improve the management of patients with severe disease, including potentially identifying those requiring early antifibrotic treatment. Given the strong link between collagen upregulation and fibrosis, these assays might also identify patients at risk of long-term fibrosis.

Studying interactions between immune cells, lung structures and the virus requires intact tissue, rarely sampled during acute respiratory infections. Consequently, our study relied on post-mortem tissue from fatal COVID-19 cases, which may overrepresent features specific to severe outcomes. While the extent to which these findings reflect in-life immunopathology is unclear, key collagen changes observed post-mortem were validated in serum samples from living patients. This underscores the value of post-mortem studies in uncovering novel disease biology and identifying predictive biomarkers, providing a framework for future viral pandemics.

## Shareable PDF

10.1183/13993003.01699-2023.Shareable1 This PDF extract can be shared freely online.
Shareable PDF

ERJ-01699-2023.Shareable

## Data Availability

All material, data generated, and code used for analysis will be made available upon publication.

## Supplementary material

**Please note:** supplementary material is not edited by the Editorial Office, and is uploaded as it has been supplied by the author. Supplementary methods ERJ-01699-2023.Methods Supplementary figures and table ERJ-01699-2023.Figures SSupplementary data file S1 ERJ-01699-2023.Data
