# Supplementary material for: Multi-omic spatial profiling reveals the unique SARS-CoV-2 lung microenvironment and collagen VI as a predictive biomarker in severe COVID-19
Source: Eur Respir J. 2025 Sep 11;66(3):2301699. doi: 10.1183/13993003.01699-2023 (PMC12441580; doi:10.1183/13993003.01699-2023)
Supplement: Supplementary file 1 [file ERJ-01699-2023.Shareable.pdf]

EUROPEAN RESPIRATORY JOURNAL
ORIGINAL RESEARCH ARTICLE
É. FENNELL ET AL.

Multi-omic spatial profiling reveals the unique SARS-CoV-2 lung microenvironment and collagen VI as a predictive biomarker in severe COVID-19

Éanna Fennell, Graham S. Taylor, Ciara I. Leahy, Aisling M. Ross, Gary Reynolds, Tracey Perry, Esther Youd, Jacob Skidmore, Radwan Ramzi Radwan Darwish, Kelly J. Hunter, Benjamin E. Willcox, Philip Jermann, Chowdhury Arif Jahangir, Arman Rahman, William M. Gallagher, Nadezhda Nikulina, Bassem Ben Cheikh, Oliver Braubach, Aaron T. Mayer, Lawrence S. Young, Dimitris Grammatopoulos, Sian Faustini, Alex Richter, Alexander C. Dowell, Tonny Venith, Onn S. Thein, Dhruv Parekh, Kylie B.R. Belchamber, David R. Thickett, Aaron Scott, Richard Attanoos, Lucia Mundo, Stefano Lazzi, Lorenzo Leoncini, Gareth Leopold, Neil Steven, Jannie Marie Bülow Sand, Morten A. Karsdal, Diana Julie Leeming, Stefan Dojcinov, Aedin Culhane, Paul G. Murray and Matthew R. Pugh

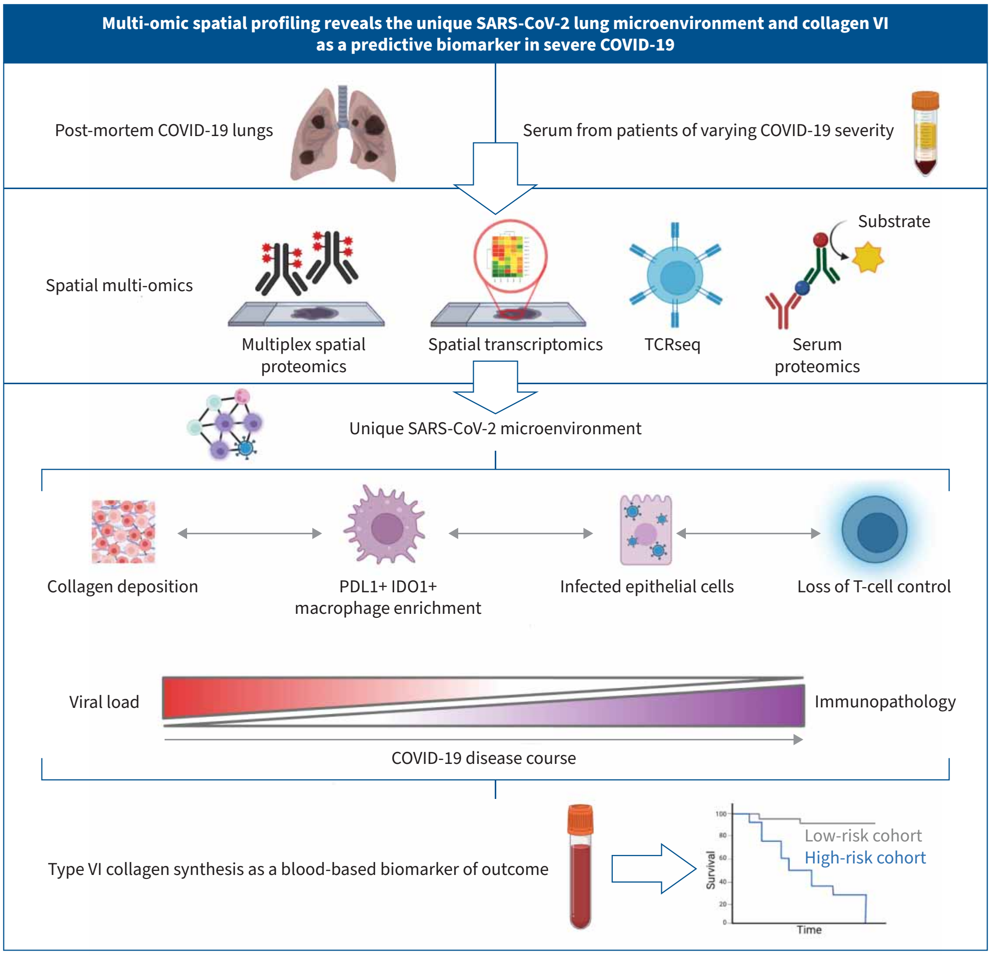

GRAPHICAL ABSTRACT Overview of the study. SARS-CoV-2: severe acute respiratory syndrome coronavirus 2; COVID-19: coronavirus disease 2019; TCR: T-cell receptor.

SHAREABLE PDF

Multi-omic spatial profiling reveals the unique SARS-CoV-2 lung microenvironment and collagen VI as a predictive biomarker in severe COVID-19

Éanna Fennell[1,2,3,24], Graham S. Taylor[4,24], Ciara I. Leahy[1,2,3], Aisling M. Ross[1,2,3], Gary Reynolds[4], Tracey Perry[4], Esther Youd[5], Jacob Skidmore[4], Radwan Ramzi Radwan Darwish[1,2,3,6], Kelly J. Hunter[7], Benjamin E. Willcox[4,7], Philip Jermann[8], Chowdhury Arif Jahangir[9], Arman Rahman[9], William M. Gallagher[9], Nadezhda Nikulina[10], Bassem Ben Cheikh[11], Oliver Braubach[12], Aaron T. Mayer[13], Lawrence S. Young[14], Dimitris Grammatopoulos[14], Sian Faustini[15], Alex Richter[4,15], Alexander C. Dowell[4], Tonny Venith[16], Onn S. Thein[17], Dhruv Parekh[17], Kylie B.R. Belchamber[17], David R. Thickett[17], Aaron Scott[17], Richard Attanoos[18], Lucia Mundo[19], Stefano Lazzi[19], Lorenzo Leoncini[19], Gareth Leopold[20], Neil Steven[4], Jannie Marie Bülow Sand[21], Morten A. Karsdal[21], Diana Julie Leeming[21], Stefan Dojcinov[20], Aedin Culhane[1,3,22,23], Paul G. Murray[1,2,3,6,25] and Matthew R. Pugh[4,25]

[1]School of Medicine, University of Limerick, Limerick, Ireland. [2]Bernal Institute, University of Limerick, Limerick, Ireland. [3]Limerick Digital Cancer Research Centre, Health Research Institute, University of Limerick, Limerick, Ireland. [4]Department of Immunology and Immunotherapy, School of Infection, Inflammation and Immunology, College of Medicine and Health, University of Birmingham, Birmingham, UK. [5]Forensic Medicine and Science, University of Glasgow, Glasgow, UK. [6]School of Medicine, Royal College of Surgeons in Ireland – Medical University of Bahrain, Adliya, Bahrain. [7]Birmingham Tissue Analytics, University of Birmingham, Birmingham, UK. [8]Department of Medical Genetics and Pathology, University Hospital Basel, Basel, Switzerland. [9]School of Biomolecular and Biomedical Science, University College Dublin, Dublin, Ireland. [10]Institute of Lung Health, Justus Liebig University Giessen, Giessen, Germany. [11]Akoya Biosciences, Marlborough, MA, USA. [12]Bruker Spatial Biology, St Louis, MO, USA. [13]Enable Medicine, Menlo Park, CA, USA. [14]Warwick Medical School, University of Warwick, Coventry, UK. [15]Clinical Immunology Service, University of Birmingham, Birmingham, UK. [16]Department of Critical Care and Anaesthesia, Queen Elizabeth Hospital Birmingham, Birmingham, UK. [17]Birmingham Acute Care Research Group, Institute of Inflammation and Ageing, University of Birmingham, Birmingham, UK. [18]Department of Pathology, University Hospital of Wales, Cardiff, UK. [19]Section of Pathology, Department of Medical Biotechnologies, University of Siena, Siena, Italy. [20]Department of Cellular Pathology, Swansea Bay University Health Board, Swansea, UK. [21]Nordic Bioscience, Herlev, Denmark. [22]Department of Data Science, Dana-Farber Cancer Institute, Boston, MA, USA. [23]Department of Biostatistics, Harvard T.H. Chan School of Public Health, Boston, MA, USA. [24]É. Fennell and G.S. Taylor contributed equally to this work. [25]P.G. Murray and M.R. Pugh contributed equally to this work.

Corresponding author: Matthew R. Pugh (M.Pugh.1@bham.ac.uk)

Check for updates

Shareable abstract (@ERSpublications)
The SARS-CoV-2 lung microenvironment is enriched for immunosuppressive macrophages in severe disease. Collagen VI deposition contributes to alveolar thickening, and detection of serum PRO-C6 is predictive for mortality in hospitalised COVID-19 patients. https://bit.ly/3E3Wh1s

Cite this article as: Fennell É, Taylor GS, Leahy CI, *et al*. Multi-omic spatial profiling reveals the unique SARS-CoV-2 lung microenvironment and collagen VI as a predictive biomarker in severe COVID-19. *Eur Respir J* 2025; 66: 2301699 [DOI: 10.1183/13993003.01699-2023].

This PDF extract can be shared freely online.

Copyright ©The authors 2025.

This version is distributed under the terms of the Creative Commons Attribution Licence 4.0.

This article has an editorial commentary: https://doi.org/10.1183/13993003.00760-2025

Abstract

Background While coronavirus disease 2019 (COVID-19) is primarily a respiratory infection, few studies have characterised the immune response to COVID-19 in lung tissue. We sought to understand the pathogenic role of microenvironmental interactions and the extracellular matrix in post-mortem COVID-19 lung using an integrative multi-omic approach.

Methods Post-mortem formalin-fixed paraffin-embedded lung tissue from fatal COVID-19 and nonrespiratory death control lung underwent multi-omic evaluation by Quantseq Bulk RNA sequencing, Nanostring GeoMx spatial transcriptomics, RNAscope, multiplex immunofluorescence and immunohistochemistry, to evaluate virus distribution, immune composition and the extracellular matrix. Markers of extracellular synthesis and breakdown were measured in the serum of 215 patients with COVID-19 and 54 healthy volunteer controls using ELISA.

Received: 5 Oct 2023
Accepted: 16 March 2025

Results We found that severe acute respiratory syndrome coronavirus 2 (SARS-CoV-2) infection was restricted to the pneumocytes and macrophages of early-stage disease. Spatial analyses revealed an immunosuppressive virus microenvironment, enriched for $PDL1^{+}IDO1^{+}$ macrophages and depleted of T-cells. Oligoclonal T-cells in COVID-19 lung showed no enrichment of SARS-CoV-2 specific T-cell receptors. Collagen VI was upregulated and contributed to alveolar wall thickening and impaired gas exchange in COVID-19 lung. Serum from COVID-19 patients showed increased levels of PRO-C6, a marker of collagen VI synthesis, predicted mortality in hospitalised patients.

Conclusions Our data refine the current model of respiratory COVID-19 with regard to virus distribution, immune niches and the role of the noncellular microenvironment in pathogenesis and risk stratification in COVID-19. We show that collagen deposition is an early event in the course of the disease.
