# Supplementary material for: Multi-omic spatial profiling reveals the unique SARS-CoV-2 lung microenvironment and collagen VI as a predictive biomarker in severe COVID-19
Source: Eur Respir J. 2025 Sep 11;66(3):2301699. doi: 10.1183/13993003.01699-2023 (PMC12441580; doi:10.1183/13993003.01699-2023)
Supplement: Supplementary file 2 [file ERJ-01699-2023.Methods.pdf]

SUPPLEMENTARY METHODS

Patient identification, tissue sampling and processing

Post-mortem (PM) tissue from patients with fatal COVID-19 and archival pre-pandemic control tissue was acquired at post-mortem examinations which were consented specifically for the LoST-SoCC study, or, underwent a hospital consent or coronial PM examination with consent for tissue use in research. Post mortems were conducted in South Wales, UK (PMs performed by GL & EY). COVID-19 PMs were conducted between April 2020 and August 2020. PM examinations and tissue acquisition were conducted under the LoST-SoCC study (IRAS 193937) following approval by the Newcastle North Tyneside 1 research ethics committee (19/NE/0336). Tissue was sampled from patients with either a positive SARS-CoV-2 swab in-life during their final illness, or from a positive swab taken at PM following a suspected SARS-CoV-2 related death. All SARS-CoV-2 patients had COVID-19/SARS-CoV02 infection stated as the cause of death on certification. Tissues were processed by formalin-fixation and paraffin-embedding (FFPE) with a minimum fixation period of 72 hours as mandated by laboratory standard operating procedures. Lung tissues were sampled from 8 COVID-19 patients, 1 Middle Eastern Respiratory Syndrome (MERS) patient, 1 Rhinovirus patient, 3 bacterial pneumonia patients and 8 non-infectious control patients. Clinical features, including timelines, were extracted from the clinical data by means of standardized proforma. Pathological features were annotated retrospectively by clinical pathologists (MRP, EY, GL, RA). Curls, slides, and tissue microarrays (TMAs) were constructed from the tissue blocks. For TMA construction, 3mm cores were extracted and embedded into an arrangement which covered less than 15mm^2 (4 x 4 tissues per TMA). Validation post-mortem lung tissue was obtained from Imperial College London Tissue

Bank, UK (Project number R20034), including 9 FFPE lung tissues from fatal COVID-19 and 3 FFPE post-mortem lung tissues from non-respiratory deaths. A further 10 COVID-19 post mortem lung tissues were obtained from the University of Sienna, Italy. The study approved by ethics committee at the University of Ferrara (CE: 342/2020/Oss/UniFe) (Supplementary data S1h,i). Post-mortem conducted on the validation cohort were conducted in 2020 prior to the introduction of the vaccine.

Serum sample cohort & processing

Serum samples from consenting participants with varying degrees of COVID-19 severity (n=215) and non-infected healthy volunteer controls (n=54) were obtained from existing collection at the University of Birmingham and Arden Biobank. Cohorts of healthy volunteers, mild/asymptomatic, and hospitalized patients were collected from the Queen Elizabeth Hospital, Birmingham, UK, the University of Birmingham, UK. Samples ethically approved for this work by the North West-Preston Research Committee on behalf of the United Kingdom Health Research Authority for the Coronavirus Immunological Analysis study (20/NW/0240). Acute ITU samples were obtained from Arden Biobank following collection from Warwick University Hospital, Coventry, UK. All samples were collected between June 2020 and August 2021, following informed consent.

EXPERIMENTAL METHODS

Chromogenic and Tinctorial Staining

Full-face 5µm thick FFPE sections were baked at 60°C for 1 hour prior to staining to ensure tissue adherence. For chromogenic immunohistochemistry, the Leica BondMax IHC protocol F (DAB) was used with the following conditions: bake and de-wax, epitope retrieval 2 for 20 minutes and collagen VI (1:100, clone EPR17072, Abcam) primary antibody incubation for 1 hour. Slides were also stained with Haematoxylin & Eosin (H&E) or picrosirius red (PSR). All slides were mounted with DPX mounting media and scanned (Aperio CS2).

PhenoCycler FUSION mIF Panel, Staining & Imaging

TMAs were sectioned at 4µm onto glass slides in preparation for PhenoCycler FUSION staining and imaging. A 38-plex antibody panel was designed against immune lineage, functional and viral markers (supplementary data file S1b). Antibodies were conjugated to unique DNA oligonucleotide barcodes in preparation for PhenoCycler staining and imaging. Glass slides were prepared, stained, and fixed as per the PhenoCycler protocol. Briefly, slides were deparaffinized in xylene and re-hydrated in decreasing concentrations of ethanol (100%, 90%, 70% & 50%). Heat Induced Epitope Retrieval (HIER) was performed in a pressure cooker at 120°C for 20 minutes in Tris-EDTA buffer (pH = 9.0). The tissue was then washed and incubated in PhenoCycler Staining Buffer (Akoya Biosciences) for 30 minutes to block non-specific binding of antibodies. Subsequently, the tissue was incubated in a cocktail of the conjugated antibodies for 3 hours at room temperature, then fixed in 4% paraformaldehyde for 10 minutes, 100% methanol for 5 minutes, fixative Reagent (Akoya Biosciences) for 20 minutes and then stored until imaging. Akoya Reporters were added to the corresponding well of a 96-well plate in preparation for imaging, based on the cycle design of the

experiment. Slides were imaged with the PhenoCycler FUSION system (from Akoya Biosciences). Autofluorescent subtraction, stitching and compression were completed in the FUSION software resulting in qptiff files. Subsequently, a more focused 10-plex panel was applied to a wider collection of lung tissues (supplementary data file S1b). The staining and imaging protocols used were identical to that of the broader biomarker panel.

Nanostring GeoMx DSP Spatial Transcriptomics

Whole sections were incubated with the CTA/COVID-19 RNA probe set and stained with fluorophore-conjugated antibodies against, CD45, EDG1 and DAPI to identify epithelial, immune, and endothelial compartments of the lung. Regions of interest (ROI) were subsequently selected based on this staining by a consultant pathologist (MP) into alveoli and blood vessel compartments. The Nanostring Cancer Transcriptome Atlas (CTA) targeted gene panel was used with the addition of COVID-19 specific genes (plus a set of negative probes). Reporter oligonucleotides were cleaved off the probes and gathered within each ROI as per the GeoMx DSP protocol. The eluted oligonucleotides were sequenced on an Illumina NextSeq 550.

QuantSeq Library Prep & Sequencing

RNA extraction was conducted using the RNeasy FFPE Kit (Qiagen 73504) according to the manufacturer’s instructions. Briefly, curls were deparaffinized in xylene and rehydrated with decreasing concentration ethanol washes. The tissue was digested in Buffer PKD with 10 μL

of proteinase K and 2 µL of β-mercaptoethanol at 56° C for 15 min followed by incubation at 80 ° C for 15 min. Samples were cooled on ice for 3 min and the digested tissue was pelleted by centrifugation. DNA was digested in DNase Booster Buffer with 10 µL of DNase I for 15 min at room temperature. The binding conditions of the sample were adjusted by adding buffer RBC and ethanol. The RNA was applied to a RNeasy spin column and washed with buffer RPE before eluting in 30 µL of RNase-free water. 5 µL of RNA was removed to determine the quality and quantity of the extracted RNA using the Agilent 4200 TapeStation and Qubit, respectively. The remaining 25 µL was utilized for QuantSeq library prep. Library prep was conducted using the Lexogen QuantSeq 3' mRNA-Seq Kit FWD for Illumina. QC was monitored by spiking in 0.5uL of SIRV to allow technical evaluation of library prep and sequencing performance. The prepared RNA was sequenced on an Illumina NextSeq 550.

RNAscope fluorescent *in-situ* hybridization

SARS-CoV-2 was detected *in situ* by multiplex fluorescence using the RNAscope® Leica Multiplex Fluorescent Assay according to the manufacturer's instructions (Advanced Cell Diagnostics (ACD), Newark, CA, USA). Probes against the sense strand of spike gene (V-nCoV2019-S, 845701, ACD) and antisense strand of the orf1ab gene (V-nCoV2019-orf1ab-sense) were used in combination with a probe designed against human *IL6*. RNAscope ISH staining was performed on the Leica Bond RX automated IHC platform. Fluorescent detection of probes was performed with TSA-based Opal fluorophores (Opal 620- spike sense probe; Opal 520 -orf1ab antisense probe). Nuclear counterstaining was performed with DAPI. Slides were imaged using the Vectra® Polaris 2.0 (Akoya Biosciences). Spectral

unmixing and autofluorescence removal was performed with InForm Tissue Analysis Software (Akoya Biosciences) with the resulting tiles stitched in QuPath.

COMET mIHC panel, staining & imaging

A 21-plex antibody panel was validated on the Lunaphore COMET to identify collagen deposition, structural markers, SARS-CoV-2, and immune cell abundance (CD45, CD45RO, COLLAGEN I, COLLAGEN III, COLLAGEN IV, COLLAGEN VI, CD68, AE1/AE3, vimentin, SMA, CD3, CD20, CD163, spike, nucleocapsid, CD15, PDL1, PD1 CD4, CD8, GPNMB). Slides were de-waxed, baked and antigen retrieved (102°C for 1 hour in a pH 9 buffer) using the PT module (Thermofisher) before a brief wash in multi-staining buffer. The slides were then loaded into the COMET platform (Lunaphore Technologies). Before antibody staining cycles commenced, images of the TRITC and Cy5 channels were captured for autofluorescence subtraction during image processing post acquisition. Primary antibodies were diluted to the desired concentrations (data file S1b) in multi-staining buffer and incubated for 4 minutes in each cycle. Secondary antibody incubation and elution was carried out for 2 minutes each. Exposure times for DAPI, TRITC and Cy5 were set at 50ms, 400ms and 200ms respectively. Secondary antibodies in the TRITC channel (mouse antibody specific) were diluted to a concentration of 1:100 while Cy5 channel secondaries (rabbit antibody specific) were diluted to1:400. DAPI images were taken in every staining cycle to ensure alignment of cycles during image processing. All antibodies were optimized using negative staining (secondary antibody only) and elution/re-staining step protocols.

TCR Sequencing

RNA extraction was conducted according to the above protocol used for QuantSeq preparation. TCR sequencing was conducted with the Oncomine TCR Beta-LR Assay (Thermo Fisher), with preparation on the Ion Chef System and sequencing on the Ion GeneStudio S5.

Serum Proteomics

Ten markers of extracellular matrix formation and degradation and a marker of neutrophil activation (supplementary table 10) were measured by competitive ELISA (Nordic Bioscience; Herlev Denmark) in serum samples of 269 patients, including varying degrees of COVID-19 severity (community, convalescent, acute hospital, acute ITU and convalescent ITU) and healthy controls. 96 well ELISA plates, precoated with streptavidin, were coated with the respective specific peptide at 20°C for 30 minutes at 300 rpm before being washed 5 times in washing buffer. 20uL of diluted sample or standard peptide was added with 100uL of peroxidase conjugated monoclonal antibodies in assay buffer. The plates were incubated for 1 hour at 20°C while being agitated at 300 rpm. Subsequently, the plates were washed 5 times with wash buffer and 100uL of TMB was added and incubated for 15 minutes in the dark at 300 rpm. 100uL of 1% H_2SO_4 was added to stop the reaction. The plates were analyzed on an ELISA reader at 450nm (650nm reference).

COMPUTATIONAL & STATISTICAL ANALYSIS

Architectural Analysis

QuPath was used to quantify the percentage of lacunae in the lung of COVID-19 patients from full-face H&E stained images. A pixel thresholder was used to detect the tissue border, with maximum hole size and maximum detection size set to 1M and 5M respectively, and the area within this border was measured. Lacunae were then detected by a second pixel classifier identifying background pixels, restricted to within the tissue border, and measured. For each tissue, the lacunar pixels were divided by the total tissue pixels and plotted for COVID-19 disease state.

Fibrosis Quantification

QuPath was used to quantify fibrosis content from picrosirius red staining. Tissue cores from scans of the TMAs were identified by pixel thresholding, and the area covered by each core was measured. The PSR stain was split into red and yellow channels by the 'Estimate stain vectors' function within QuPath. A second pixel thresholder was used to identify positive pixels for the red channel (>0.3) and these were divided by the total tissue pixels to get the fibrosis content within each core. Fibrosis content was subsequently plotted as a function of disease state and disease course within the COVID-19 cohort.

LASSO model

A LASSO regression model was used to calculate the genes most predictive of fibrosis, disease length and lacunar space. These features were imported into python with matched

bulk RNA sequencing from the lungs of the same patients. The linear model LASSO function (scikit-learn) was used to fit a LASSO model to this data and the output was ordered for genes with the largest residuals.

Multiplex immunohistochemistry image pre-processing

Raw TIFF images (qptiff/ome.tiff) were ran through a bespoke pre-processing pipeline comprising de-arraying, pseudo-membrane marker generation, cellular segmentation, and feature extraction. CD45, aSMA, PanCK and CD15 were merged to create a pseudo-membrane marker for segmentation. Segmentation was completed on a per-core basis on the stitched DAPI and pseudo-membrane marker channels using CellSeg [1]. Segmentation masks and their borders for each core were saved. FCS files were generated using the segmentation masks with the 'regionprops' function within Python, exporting the average intensity of each channel and morphological features of each cell (supplementary data file S1e).

Phenotyping & virus identification

All numerical cellular data were imported into MISSILe for phenotypic and spatial analyses. Cells were first filtered based on size. For the PhenoCyler FUSION images, cells were first screened into four categories by CELESTA, endothelial, epithelial, immune and aSMA+ based on CD34, pan-cytokeratin, CD45 and aSMA respectively (see supplementary data file S1f for CELESTA parameters) [2]. The immune cells were then clustered using PhenoGraph (FastPG) to identify the main subsets [3]. All cells were then screened for positivity for functional markers (Ki67, PDL1, HLA-DR, etc.) using silhouette clustering of the histograms

on by core basis, including positivity for SARS-CoV-2 spike and nucleocapsid proteins. Only cells identified as positive for both viral markers were labelled as infected.

Virus microenvironment quantification

To quantify the immediate immune microenvironment of SARS-CoV-2 infected lung epithelium, cellular abundances were recorded as a function of distance from the infected cells. The nn2 function (RANN library) was used to calculate the N (where N = 10, 20, 40, 80, 100, 200, 400, & 800) number of cells closest to infected cells. The phenotype of each cell was then calculated as a percentage of the total cells and total immune cells in each bin (N) and plotted. For fair comparisons, the microenvironments of virus infected epithelium were compared to microenvironments of non-infected epithelium, with the same number of cells, within the same samples.

Collagen content quantification on multiplex immunohistochemistry

To assess the collagen content of COVID-19 lungs, collagen IV and VI protein channels from the COMET were assessed. To calculate the total tissue area, the intensity of all channels was averaged. Otsu thresholding determined positive tissue pixels and the total number of these was measured. Collagen 4 and 6 channels were also run through Otsu thresholding to define positive pixels for both and the total of each were subsequently divided by total tissue area. This was conducted on a by core basis and plotted as a function of disease state.

Quantification of collagen VI chromogenic immunohistochemistry

To assess the collagen content within alveolar walls, image analysis was performed using the QuPath software. Regions of interest (ROIs) corresponding to the alveolar walls were manually annotated using the brush tool to properly delineate wall from background. The total area of the alveolar wall was computed from the annotated regions.

A pixel classifier was created with a threshold value of 0.75, set based on the morphological appearance of collagen within the stained sections by trial and error. Pixels with intensity values greater than the threshold value were classified as collagen-positive, while those with lower intensity values were classified as collagen-negative. Both alveolar wall area and collagen-positive areas were quantified in each section (px^2). The ratio of collagen to alveolar wall area was then calculated, providing a standardized metric across all sections.

Graphical analyses were performed in R using the *ggplot2* package, where three primary metrics were examined: (1) collagen area, (2) alveolar wall area, and (3) the ratio between (1) and (2). The stained sections were split into three groups: normal samples, London COVID samples, and Italian COVID samples. Box plots were used to visualize the data.

Spatial Transcriptomic Pre-processing & Analysis

Fastq files from the GeoMx DSP sequencing run were concatenated and processed through the Nanostring GeoMx NGS Pipeline to generate Digital Count Conversion (DCC) files. DCC files were imported into the Nanostring interactive data analysis and visualisation

software. Regions failing QC were excluded from the analysis and Q3 normalisation was subsequently performed. Gene count and metadata files were exported in csv format and imported with annotated metadata files into R. Gene set expression was calculated by taking the addition of genes within selected gene sets per region.

RNAscope FISH Analysis

Stitched tiff images were imported into QuPath. TMAs were de-arrayed and linked to tissue type and disease group. Cells were segmented and detection measurements were exported as a CSV file. These files were imported into R and silhouette histogram clustering was used to assign positivity of each channel to every cell. Cells identified as SARS-CoV-2 infected were interrogated for the underlying cell type by pan-cytokeratin protein expression. On a per patient basis, the number of infected cells were correlated with mIHC and bulk mRNA virus data.

QuantSeq RNA Alignment & Analysis

Fastq files from each flow cell were concatenated for each sample replicate. RNA quality control of the fastq files was assessed with FASTQC [4]. According to Lexogen recommendations of the 3’ assay, the adapter contamination, polyA read through and low-quality tails were trimmed using bbduk. The SARS-CoV-2 genome files from NCBI were merged with the GRCh38 Homo sapiens genome, and a new reference was created with the STAR aligner [5]. Alignment was then conducted using STAR, with duplicate reads

subsequently removed with PICARD. The resulting BAM files were indexed using SAMtools and counted using HTSEQ-count [6, 7]. The count files were then imported into R and DESeq2 [8], where they were normalised using the median of ratios. Gene set enrichment analysis was conducted with the GSEA program from the Broad Institute and heatmaps were visualized using the ComplexHeatmap library [9, 10]. Gene set expression was calculated by taking the addition of genes within selected gene sets per region.

Differential Gene Expression & Pathway Analysis

Differential gene expression testing of Nanostring GeoMx DSP and QuantSeq RNA data was conducted with DeSeq2 and plotted with Enhanced Volcano. EnrichR was used to calculate gene ontologies with significant up-/down-regulated genes with a p-value of less than 0.05 and plotted [11].

TCR sequencing analysis

TCR beta sequences from lung tissue samples were quality controlled, removing reads shorter than 6 amino acids in length. TCR beta sequences reported to be specific for pathogens were obtained from the VDJDB [12] and McPAS databases [13]. Additional SARS-CoV-2 specific TCR sequences were obtained from [14]. Sequencing matching was performed using the GREP function within R v4.0.3.

Serum Proteomic Analysis

Serum proteomics data were imported into R and organized into assay results per sample. Each assay was plotted against disease group and significance was assessed by the Wilcoxon statistical test. Samples with sufficient appropriate clinical data were plotted against time post symptom onset/evidence of disease and survival curves were plotted, initially for acute hospital and acute ITU cases separately, with the survminer package in R. Subsequently, the acute hospital and ITU cases were combined for a Cox-proportional hazards test with age, sex and ethnicity used as confounding variables.

References

[1] Lee, M.Y., Bedia, J.S., Bhate, S.S., Barlow, G.L., Phillips, D., Fantl, W.J., Nolan, G.P., and Schurch, C.M. (2022). CellSeg: a robust, pre-trained nucleus segmentation and pixel quantification software for highly multiplexed fluorescence images. BMC Bioinformatics *23*, 46.

[2] Zhang, W., Li, I., Reticker-Flynn, N.E., Good, Z., Chang, S., Samusik, N., Saumyaa, S., Li, Y., Zhou, X., Liang, R., *et al.* (2022). Identification of cell types in multiplexed in situ images by combining protein expression and spatial information using CELESTA. Nat Methods *19*, 759-769.

[3] Bodenheimer, T., Halappanavar, M., Jefferys, S., Gibson, R., Liu, S., Mucha, P.J., Stanley, N., Parker, J.S., and Selitsky, S.R. (2020). FastPG: fast clustering of millions of single cells. BioRxiv.

[4] Andrews, S. (2010). FastQC: A Quality Control Tool for High Throughput Sequence Data.

[5] Dobin, A., Davis, C.A., Schlesinger, F., Drenkow, J., Zaleski, C., Jha, S., Batut, P., Chaisson, M., and Gingeras, T.R. (2013). STAR: ultrafast universal RNA-seq aligner. Bioinformatics *29*, 15-21.

[6] Anders, S., Pyl, P.T., and Huber, W. (2015). HTSeq--a Python framework to work with high-throughput sequencing data. Bioinformatics *31*, 166-169.

[7] Putri, G.H., Anders, S., Pyl, P.T., Pimanda, J.E., and Zanini, F. (2022). Analysing high-throughput sequencing data in Python with HTSeq 2.0. Bioinformatics *38*, 2943-2945.

[8] Love, M.I., Huber, W., and Anders, S. (2014). Moderated estimation of fold change and dispersion for RNA-seq data with DESeq2. Genome Biol *15*, 550.

[9] Gu, Z., Eils, R., and Schlesner, M. (2016). Complex heatmaps reveal patterns and correlations in multidimensional genomic data. Bioinformatics *32*, 2847-2849.

[10] Subramanian, A., Tamayo, P., Mootha, V.K., Mukherjee, S., Ebert, B.L., Gillette, M.A., Paulovich, A., Pomeroy, S.L., Golub, T.R., Lander, E.S., *et al.* (2005). Gene set enrichment analysis: a knowledge-based approach for interpreting genome-wide expression profiles. Proc Natl Acad Sci U S A *102*, 15545-15550.

[11] Chen, E.Y., Tan, C.M., Kou, Y., Duan, Q., Wang, Z., Meirelles, G.V., Clark, N.R., and Ma'ayan, A. (2013). Enrichr: interactive and collaborative HTML5 gene list enrichment analysis tool. BMC Bioinformatics *14*, 128.

[12] Goncharov, M., Bagaev, D., Shcherbinin, D., Zvyagin, I., Bolotin, D., Thomas, P.G., Minervina, A.A., Pogorelyy, M.V., Ladell, K., McLaren, J.E., *et al.* (2022b). VDJdb in the pandemic era: a compendium of T cell receptors specific for SARS-CoV-2. Nat Methods *19*, 1017-1019.

[13] Tickotsky, N., Sagiv, T., Prilusky, J., Shifrut, E., and Friedman, N. (2017). McPAS-TCR: a manually curated catalogue of pathology-associated T cell receptor sequences. Bioinformatics *33*, 2924-2929.

[14] Nolan, S., Vignali, M., Klinger, M., Dines, J.N., Kaplan, I.M., Svejnoha, E., Craft, T., Boland, K., Pesesky, M., Gittelman, R.M., *et al.* (2020). A large-scale database of T-cell receptor beta (TCRβ) sequences and binding associations from natural and synthetic exposure to SARS-CoV-2. Res Sq.
