# Supplementary material for: Multi-omic spatial profiling reveals the unique SARS-CoV-2 lung microenvironment and collagen VI as a predictive biomarker in severe COVID-19
Source: Eur Respir J. 2025 Sep 11;66(3):2301699. doi: 10.1183/13993003.01699-2023 (PMC12441580; doi:10.1183/13993003.01699-2023)
Supplement: Supplementary file 3 [file ERJ-01699-2023.Figures.pdf]

SUPPLEMENTARY FIGURES

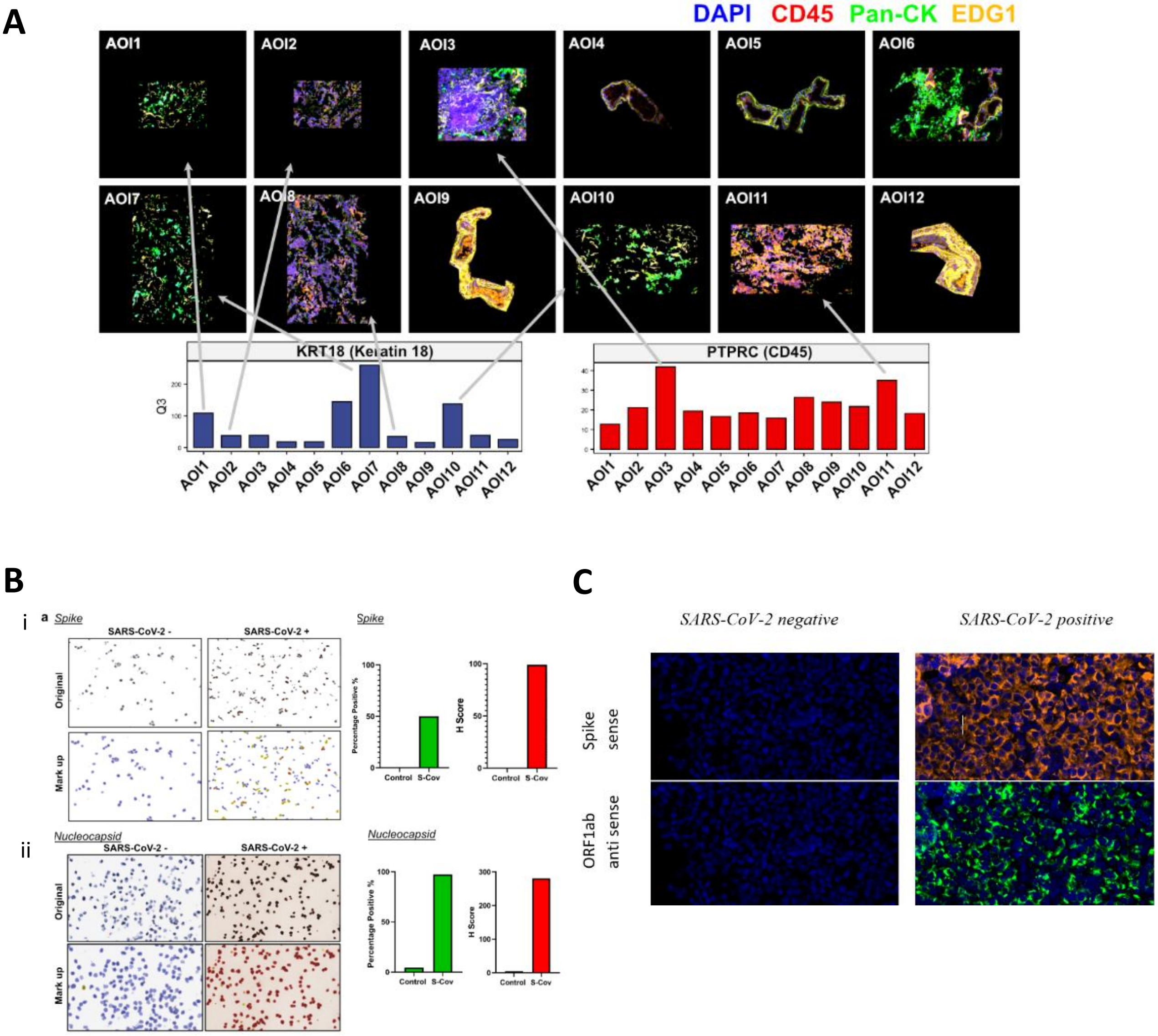

Figure S1. Experimental and reagent validation. (A) Validation of Nanostring GeoMx DSP showing high levels of expression of keratin 18 by gene expression (RNA) in areas of interest (AOI) which stained strongly for pan-cytokeratin (AE1/AE3 IHC), and high levels of PTPRC (CD45) gene expression (RNA) in AOIs which stained strongly for immune cells (CD45 IHC). (B) SARS-CoV-2 antibody validation of spike (40150-T62-COV2; Sino Biological) and nucleocapsid (40143-MM08; Sino Biological) proteins. (i) Cytospin staining and signal quantification of spike protein on transfected Vero cells. (ii) Cytospin staining and signal quantification of nucleocapsid protein on transfected Vero cells. (C). Validation of SARS-CoV-2 RNAscope probes. Epithelial cell lines with and without SARS-CoV-2 virus infection were established. The cell lines were pelleted, formalin-fixed, and paraffin-embedded (FFPE). FFPE sections for infected and non-infected cell lines were stained with spike sense and ORF1ab anti-sense RNAscope probes. Infected cell line sections showed strong signals for both probes, indicative of replicative infection. No signal was seen in the uninfected cell lines.

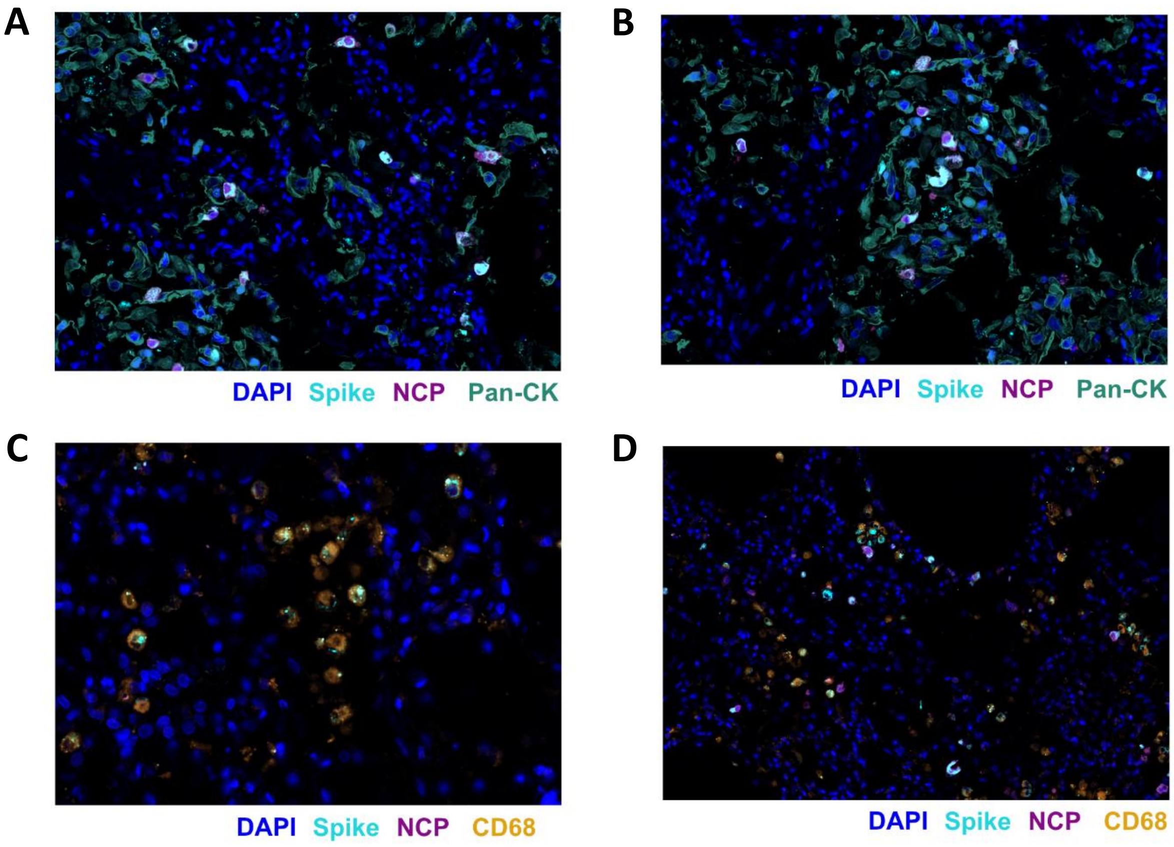

Figure S2. SARS-CoV-2 mIHC detection. Additional representative images of dual antibody-based detection of SARS-CoV-2 by mIF showing (A & B) infection of pan-cytokeratin positive cells and (C & D) SARS-CoV-2 co-localizing with CD68+ macrophages.

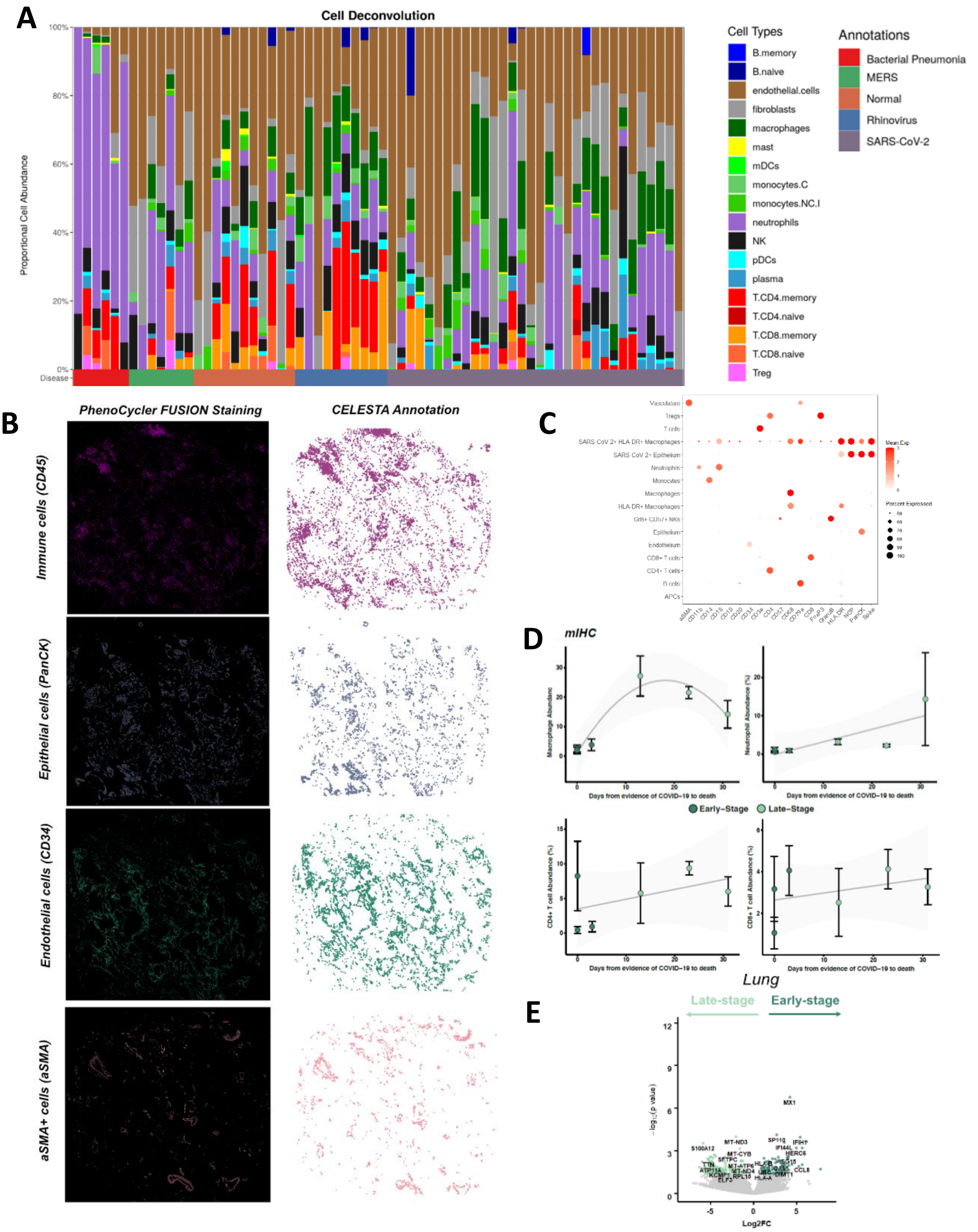

Figure. S3. Immune cell phenotyping and composition. (A) Deconvoluted GeoMX data showing relative cellular abundances for each region of interest (ROI). (B) CELESTA cell phenotype annotation validation of four broad phenotypes from PhenoCycler Fusion staining. (C) Cluster marker expression dot plot showing the mean expression (Mean.Exp) and percentage of cells expressing each marker per cluster from the PhenoCycler Fusion expanded panel. (D) Immune cell abundances of macrophages, neutrophils, CD4+ T-cells and CD8+ T-cells against the length of illness in COVID-19 post-mortem lungs. All subsets increased with the duration of illness despite virus clearance. (E) Differential gene expression analysis from bulk RNA sequencing of early- vs. late-stage COVID-19.

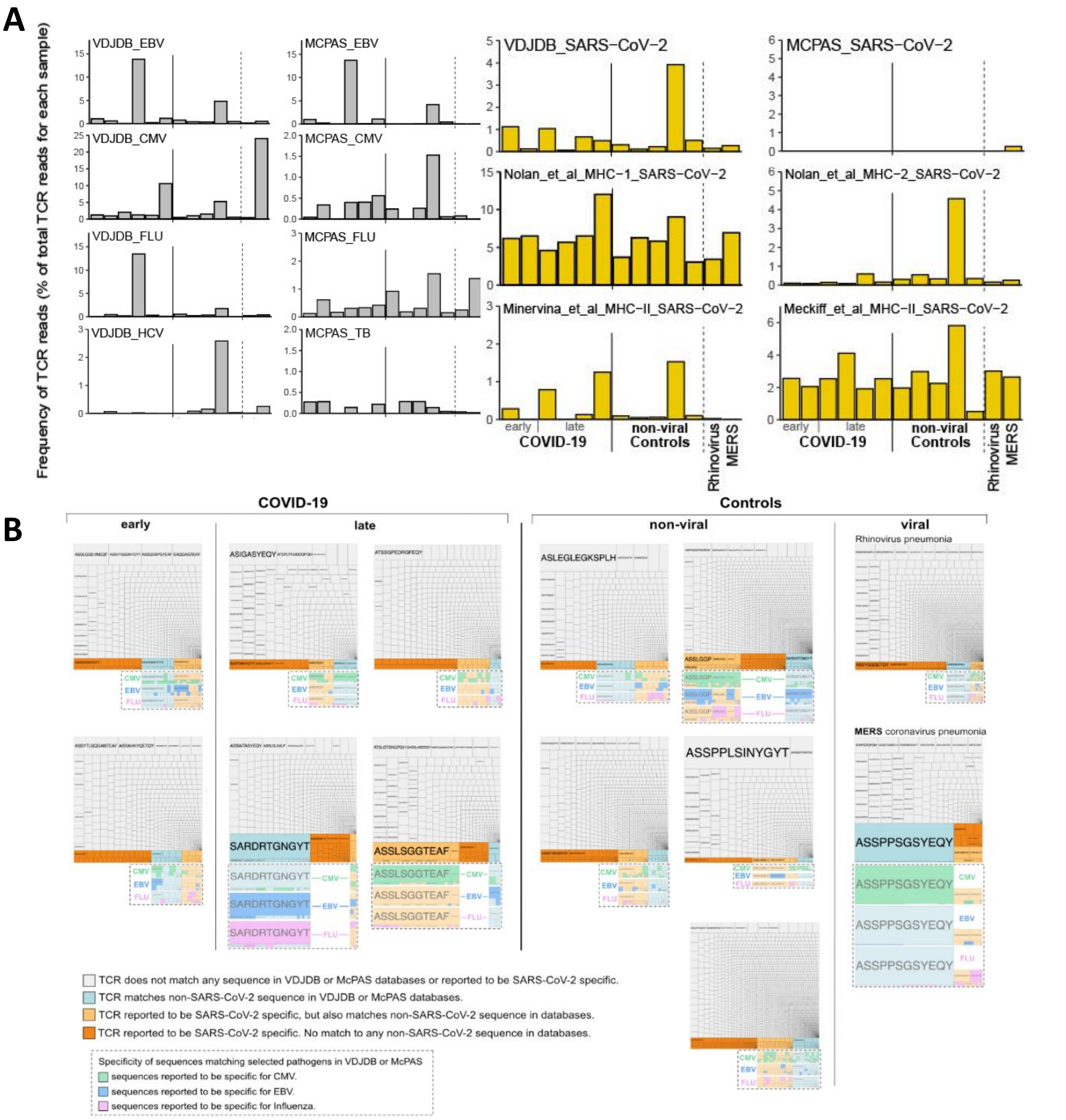

Figure S4. **TCR sequencing extended data**. (A) Frequencies of lung TCR-Vbeta sequences matching different SARS-CoV-2 TCR datasets. Yellow bars show the percentage of lung tissue TCR reads from each COVID-19 or control patient that match five different SARS-CoV-2 TCR datasets: (Meckiff et al., 2020; Minervina et al., 2021; Nolan et al., 2020) The data from Nolan *et al*. is sub-divided into MHC class-I and MHC class-II TCR sequences. Also shown are the percentage of lung tissue TCR reads matching TCRs in the VDJDB or McPAs databases reported to be specific for selected pathogens (grey bars). (B) Treemaps of lung TCR-Vbeta sequences from patients with COVID-19 and control patients. Plots show the relative frequencies of individual TCR-beta clonotypes detected in each patient's lung tissue sample. TCR sequences from patients that were shorter than 7 amino acids were removed prior to analysis. Colours in each large panel show whether each TCR matches sequences reported to be specific for SARS-CoV-2 but not any other antigen (dark orange), SARS-CoV-2 but also another non-SARS-CoV-2 antigen (light orange), or any other antigen (light blue). Sequences that did not match are coloured grey. The smaller panel replots the sequences matching non-SARS-CoV-2 specific TCRs with colours indicating those matching TCR sequences in the VDJDB and McPAS sequences reported to be specific for Epstein-Barr virus (EBV), Cytomegalovirus (CMV) or Influenza A (FLU). SARS-CoV-2-specific TCR-Vbeta sequences were obtained from the VDJDB and McPAS databases and the following references: (Meckiff et al., 2020; Minervina et al., 2021; Nolan et al., 2020). Non-SARS-CoV-2 specific sequences were obtained from the VDJDB and McPAS databases.

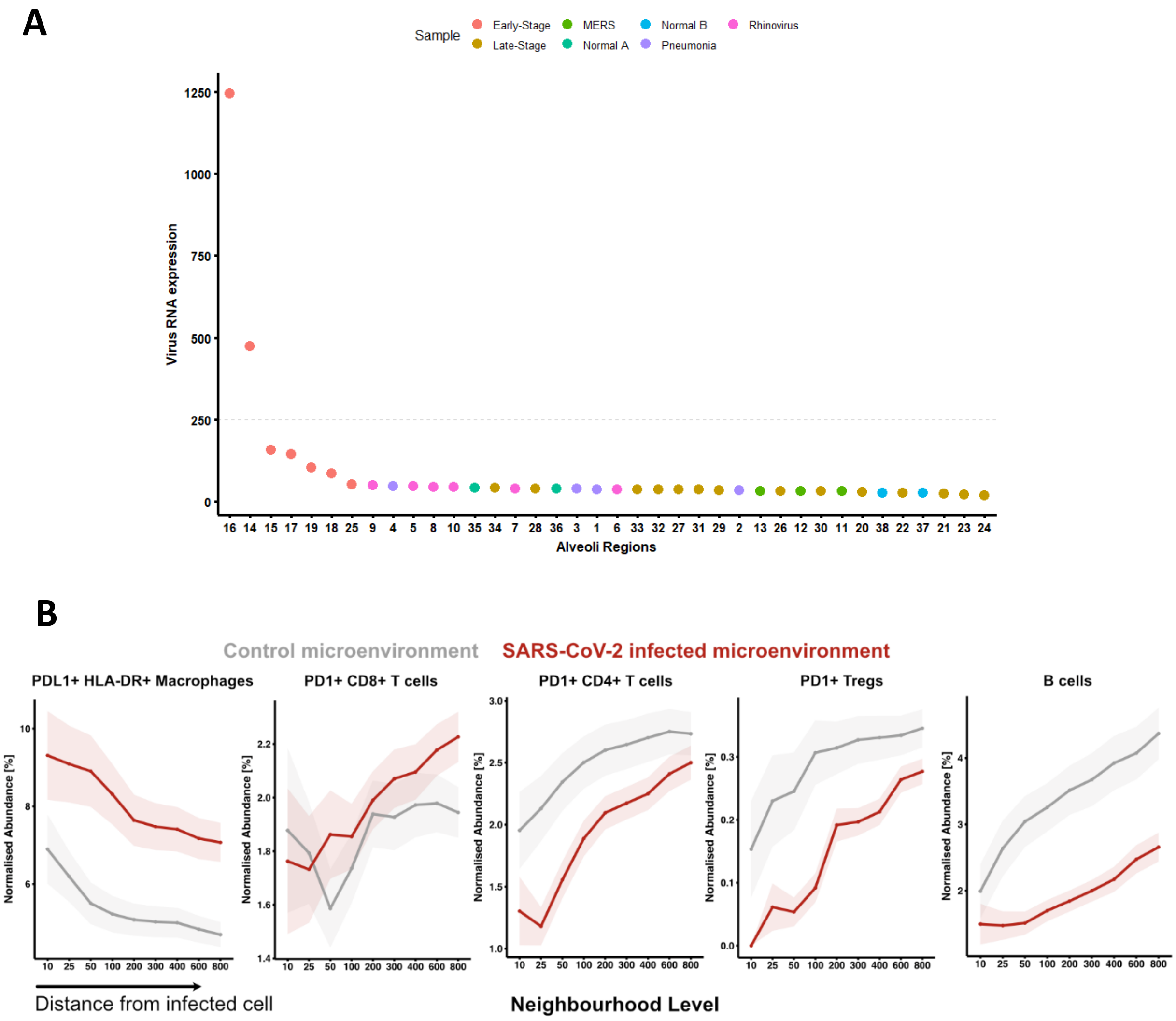

Figure S5. Extended virus neighborhood analysis data. (A) Virus high and low area of interest selection: Ordered SARS-CoV-2 expression of lung alveoli (colored by disease state) by Nanostring GeoMx DSP with a horizontal line at the positivity cut-off. (B) Extended characterization of the immediate microenvironment of SARS-CoV-2 infected epithelium.

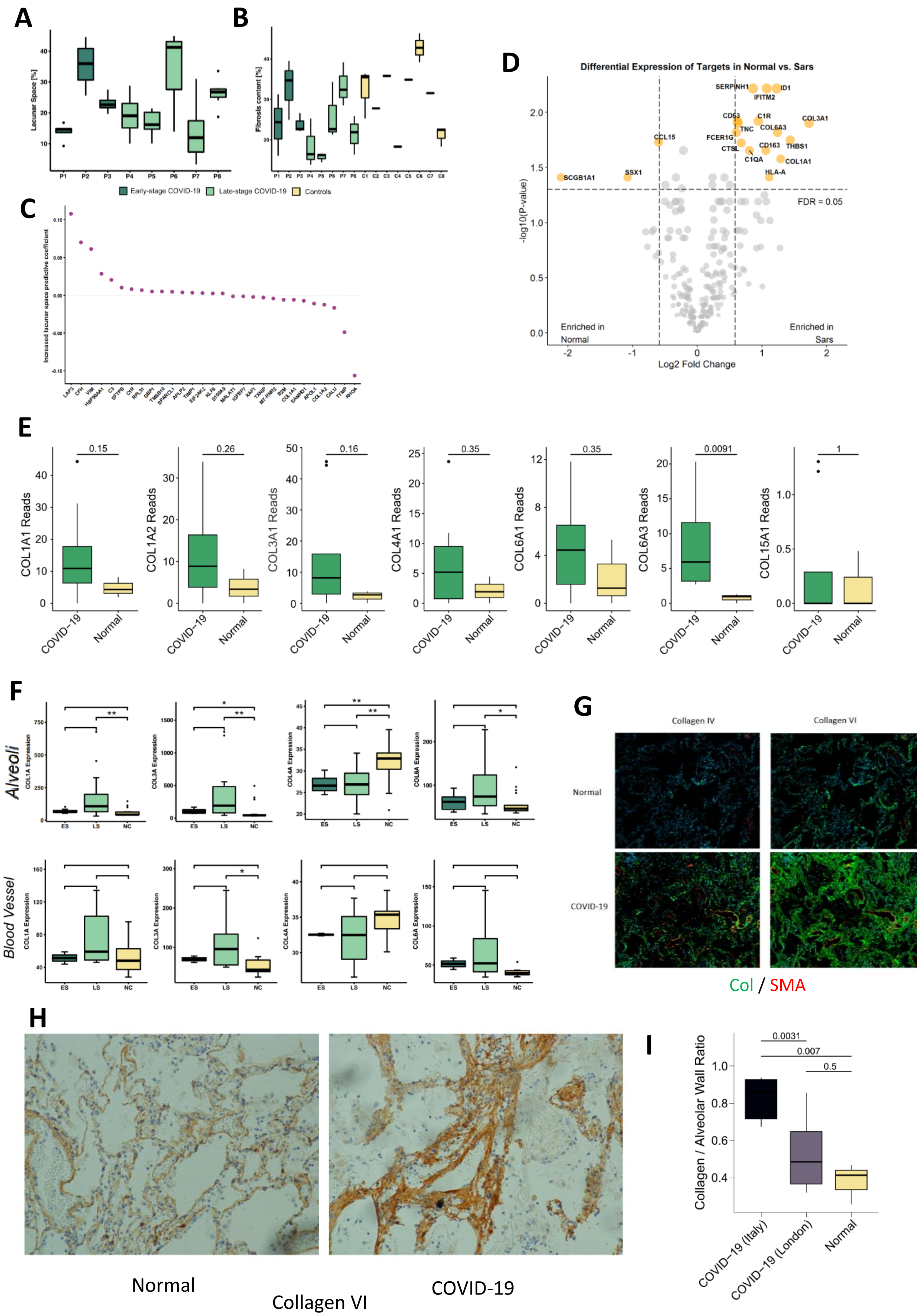
A
Lacunar Space [%]
P1 P2 P3 P4 P5 P6 P7 P8
B
Fibrosis content [%]
P1 P2 P3 P4 P5 P6 P7 P8 C1 C2 C3 C4 C5 C6 C7 C8
Early-stage COVID-19
Late-stage COVID-19
Controls
C
Increased lacunar space predictive coefficient
D
Differential Expression of Targets in Normal vs. Sars
-log10(P-value)
Log2 Fold Change
SERPINH1
ID1
IFITM2
CD53
C1R
COL3A1
TNC
COL6A3
CCL15
FCER1G
CTSL
CD163
THBS1
C1QA
COL1A1
SSX1
HLA-A
SCGB1A1
FDR = 0.05
Enriched in Normal
Enriched in Sars
E
COL1A1 Reads
COL1A2 Reads
COL3A1 Reads
COL4A1 Reads
COL6A1 Reads
COL6A3 Reads
COL15A1 Reads
0.15
0.26
0.16
0.35
0.35
0.0091
1
COVID−19
Normal
F
Alveoli
Blood Vessel
COL1A Expression
COL3A Expression
COL4A Expression
COL6A Expression
ES LS NC
G
Collagen IV
Collagen VI
Normal
COVID-19
Col / SMA
H
Normal
Collagen VI
COVID-19
I
Collagen / Alveolar Wall Ratio
0.0031
0.007
0.5
COVID−19 (Italy)
COVID−19 (London)
Normal

Figure S6. Extended collagen data: (A) Lacunar space & (B) fibrosis content per patient. (C) Genes predictive of lacunar space from LASSO model. (D) Volcano plot showing upregulation of collagen genes in COL1A1, COL3A1 and COL6A3 in COVID-19 lung compared to normal control (all areas of interest combined). (E) Validation of RNAseq collagen expression in a separate cohort (COVID-19 n=10; Normal n=10). (F) Nanostring GeoMX gene expression in the alveoli and blood vessels of COVID-19 cases and controls by Nanostring GeoMx DSP. (G) COMET mIHc showing COLIV and COLVI staining in COVID-19 lung vs control. (H) Chromogenic single-plex IHC staining for collagen VI in COVID-19 lung vs control, showing thickened alveolar walled and fibrillar collagen VI deposition in COVID-19 lung compared to normal lung, which showed a thin "tram-track" bi-layer of collagen VI in the alveolar wall. (I) Quantification of collagen VI deposition to alveolar wall size in COVID-19 (across two cohorts) and Normal lungs.

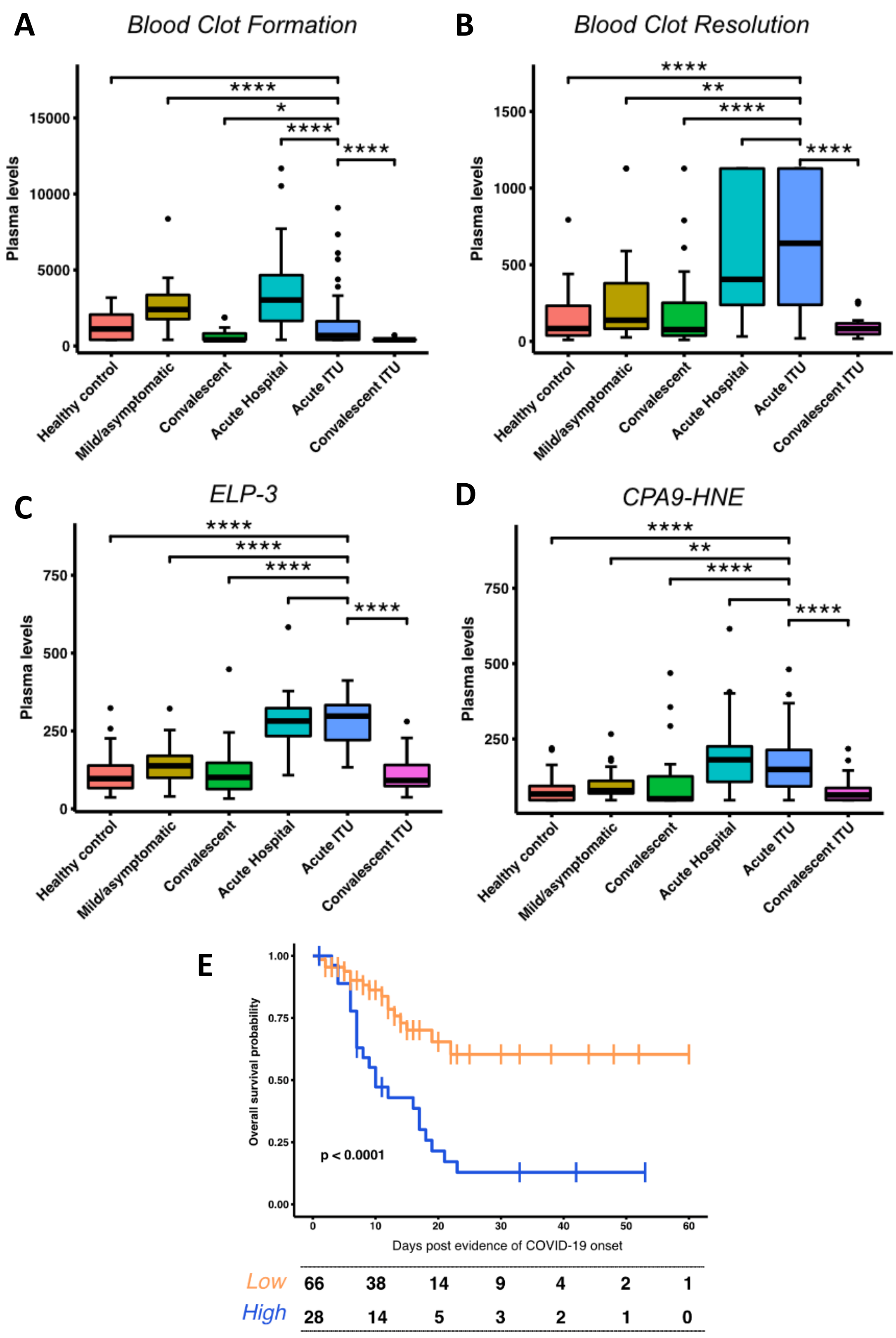

Figure S7. Extended ELISA panel results and overall survival for all hospitalized patients. ELISA results for blood clot formation (A), blood clot degradation (B) markers of neutrophil activation ELP-3 (C) and CPA9-HNE (D) measured by ELISA. (E) Survival of all hospitalized COVID-19 patients (acute hospital & acute ITU cohorts).

SUPPLEMENTARY TABLE

Cohort	SARS-CoV-2 Post mortem		
Subgroup	*All*<br><br>*n/8 (%)*	*Early stage acute Death<7days*<br>*n/3 (%)*	*Late stage acute Death ≥ 7 days*<br>*n/5 (%)*
**Age**			
50-59	1 (13)	0 (0)	1 (20)
60-69	2 (25)	0 (0)	2 (40)
70-79	2 (25)	1 (33)	1 (20)
80-89	3 (38)	2 (66)	1 (20)
**Sex**			
Male	3 (38)	1 (33)	2 (40)
Female	5 (63)	2 (66)	3 (60)
**Ethnicity**			
Caucasian	8 (100)	3 (100)	5 (100)
**Body mass index**			
Underweight	1 (13)	0 (0)	1 (20)
Normal	2 (25)	1 (33)	1 (20)
Overweight	4 (50)	2 (66)	2 (40)
Obese	1 (13)	0 (0)	1 (20)
**Clinical history**			
Hypertension	3 (38)	1 (33)	2 (40)
Congestive heart disease	1 (13)	1 (33)	0 (0)
Diabetes	2 (25)	1 (33)	1 (20)
Dementia	4 (50%)	3 (100)	1 (20)
COPD/Asthma	3 (38)	1 (33)	2 (40)
Immunosuppression	1 (13)	0 (0)	1 (20)
**COVID-19 Presentation**			
Cough	2 (25)	1 (33)	1 (20)
Shortness of breath	4 (50)	0 (0)	5 (80)
Fever	1 (13)	0 (0)	1 (20)
Collapse	3 (38)	3 (100)	0 (0)
**COVID-19 course**			
Community death	3 (38)	3 (100)	0 (0)
Hospital death	5 (63)	0 (0)	5 (100)
Non-invasive ventilation	1 (13)	0 (0)	1 (20)
Invasive ventilation	1 (13)	0 (0)	1 (20)
**Macroscopic pathology lung**			
Heavy lungs (right>720g;left>675g)	5 (63)	2 (66)	3 (60)
Bronchial mucositis	5 (63)	3 (100)	2 (40)
Consolidation	6 (75)	2 (66)	4 (80)
Lobar pneumonia	1 (13)	1 (33)	0 (0)
**Microscopic pathology lung**			
Diffuse alveolar damage	8 (100)	3 (100)	5 (100)
Organising pneumonia	1 (13)	0 (0)	1 (20)
Microthrombi	4 (50)	1 (33)	3 (60)
Lymphocytic vasculitis	3 (38)	0 (0)	3 (60)
Focal secondary infection	3 (38)	0 (0)	3 (60)
Multifocal or widespread secondary infection	2 (25)	0 (0)	2 (40)
Bacterial secondary infection	4 (50)	0 (0)	4 (80)
Fungal secondary infection (mucormycosis)	1 (13)	0 (0)	1 (20)
Fat embolus	1 (13)	1 (33)	0 (0)

Supplementary Table S1. Clinicopathological features of the post-mortem cohort.
